# The non-linear association between creatinine-to-albumin ratio and medium-term mortality in patients with sepsis accompanied by acute kidney injury in the intensive care unit: a retrospective study based on the MIMIC database and external validation

**DOI:** 10.3389/fcimb.2025.1602921

**Published:** 2025-12-05

**Authors:** Jiaqi Lou, Ziyi Xiang, Xiaoyu Zhu, Jingyao Song, Shengyong Cui, Jiliang Li, Sida Xu, Neng Huang, Xin Le, Youfen Fan, Guoying Jin

**Affiliations:** 1 Burn Department, Ningbo No. 2 Hospital, Ningbo, Zhejiang, China; 2 Institute of Pathology, Faculty of Medicine, University of Bonn, Bonn, Germany; 3 Health Science Center, Ningbo University, Ningbo, Zhejiang, China; 4 School of Mental Health, Wenzhou Medical University, Wenzhou, Zhejiang, China

**Keywords:** sepsis, acute kidney injury, creatinine to albumin ratio, prognostic biomarker, mortality, critical care medicine, inflammation, renal dysfunction

## Abstract

**Objective:**

This study aimed to evaluate the prognostic value of the Creatinine to Albumin Ratio (CAR) in predicting 30-day mortality in patients with sepsis complicated by acute kidney injury (AKI).

**Methods:**

This retrospective cohort study utilized the MIMIC-IV database (v2.2) to analyze data from 2,712 adult patients diagnosed with sepsis and AKI. External validation was performed using a single-center cohort of 412 patients from Ningbo No. 2 Hospital (January 2022–December 2024). Patients were stratified into quartiles based on CAR values. The primary outcome was 30-day mortality, analyzed using Kaplan-Meier survival curves, Cox proportional hazards regression models, and restricted cubic spline (RCS) analysis. Subgroup analyses were conducted to explore the consistency of CAR’s prognostic utility across various patient demographics and clinical characteristics. Infection types were categorized based on ICD-9/10 codes into pulmonary, abdominal, urinary tract, bloodstream, and other infections. Patients with chronic kidney disease (CKD) were excluded to minimize confounding by pre-existing renal impairment.

**Results:**

Among 2,712 included patients, those in the highest CAR quartile (Q4) had the lowest survival probability. Kaplan-Meier analysis showed significant differences in mortality across quartiles (log-rank P<0.001). In fully adjusted Cox models that included newly incorporated metabolic and hemodynamic variables (electrolytes, lactate, and vasopressors use), both continuous CAR (Hospital mortality: HR = 1.16, P = 0.048; ICU mortality: HR = 1.18, P = 0.044) and Q4 (Hospital mortality: HR = 1.72, P<0.001; ICU mortality: HR = 1.61, P<0.001) were independently associated with increased mortality risk. RCS analysis revealed a J-shaped relationship with ICU mortality, with an inflection point at CAR = 1.2 mg/dL. External validation confirmed CAR’s prognostic value for 30-day mortality, with consistent associations observed in the validation cohort (Hospital mortality: HR = 1.21, 95% CI: 1.02–1.43, P = 0.032). Time-dependent ROC analysis showed strong early predictive accuracy (7-day AUC = 0.75). Subgroup analyses confirmed CAR’s robust prognostic value, which remained consistent across different infection types and was particularly pronounced in older, obese, and mechanically ventilated patients.

**Conclusion:**

CAR is an independent predictor of 30-day mortality in sepsis-associated AKI, with both continuous measurements and the highest quartile (Q4) demonstrating significant associations after comprehensive adjustment for metabolic and hemodynamic parameters. The identified threshold (CAR = 1.2 mg/dL) enhances its clinical utility. While CAR provides independent prognostic information, its modest effect sizes suggest it should be used as a complementary tool rather than a standalone predictor. CAR’s simplicity and accessibility make it a valuable adjunct for risk stratification in this high-risk population.

## 1 Background

Sepsis and its most severe form, septic shock, remain among the leading causes of mortality in critically ill patients worldwide. Despite advances in critical care medicine, the mortality rate associated with septic shock remains alarmingly high. Acute kidney injury (AKI), a frequent complication of sepsis, further exacerbates patient morbidity and mortality. AKI in the context of sepsis is associated with impaired renal function, prolonged hospital stays, increased need for renal replacement therapy (RRT), and higher mortality rates (Dong et al., 2024; Wu et al., 2024). Identifying reliable biomarkers that can predict prognosis and guide therapeutic decisions in patients with sepsis complicated by AKI is therefore a critical unmet need in clinical practice.

Current prognostic tools for sepsis patients with AKI, such as the Sequential Organ Failure Assessment (SOFA) score (Tibi et al., 2023) and Acute Physiology and Chronic Health Evaluation (APACHE) score (Zhao et al., 2023), provide valuable insights into disease severity and patient outcomes. However, these tools often rely on complex calculations and multiple variables, which may limit their utility in rapidly evolving clinical scenarios. Moreover, they do not specifically account for the interplay between renal dysfunction and systemic inflammation, which is central to the pathophysiology of sepsis-induced AKI (He FF. et al., 2022). As a result, there is a pressing need for simpler, more accessible biomarkers that can complement existing tools and enhance prognostic accuracy.

In recent years, emerging research has highlighted the potential of combining laboratory parameters to develop novel prognostic indices. Among these, the Creatinine to Albumin Ratio (CAR) has garnered attention as a promising candidate. CAR integrates two key biomarkers: serum creatinine, a marker of renal function, and serum albumin, a reflection of nutritional status and systemic inflammation. Both parameters are routinely measured in clinical practice, making CAR a readily available and cost-effective tool. Preliminary studies have suggested that CAR may correlate with disease severity and outcomes in various clinical settings, including chronic kidney disease, heart failure, and cancer (Tewari et al., 2024; Claudel and Verma, 2025; Rashidian et al., 2025). However, its utility in predicting prognosis in patients with sepsis complicated by AKI remains underexplored.

The pathophysiology of sepsis and AKI involves a complex interplay of systemic inflammation, endothelial dysfunction, and metabolic derangements (Marzouk et al., 2025). Elevated serum creatinine reflects impaired glomerular filtration, while hypoalbuminemia may result from increased capillary permeability, reduced synthesis, or increased catabolism during systemic inflammation (Zhang Q. et al., 2024). The combination of these two parameters in CAR may therefore provide a more comprehensive assessment of both renal dysfunction and systemic inflammatory burden. This dual perspective could enhance the ability to predict patient outcomes, particularly in high-risk populations such as those with sepsis and AKI.

Given the high mortality associated with sepsis and AKI, and the limitations of existing prognostic tools, this study aimed to evaluate the prognostic value of CAR in predicting 30-day mortality in patients with sepsis complicated by AKI. By leveraging the rich clinical data available in the MIMIC-IV database (Lou et al., 2024), we sought to explore whether CAR could serve as an independent predictor of mortality and whether its association with outcomes varied across different patient subgroups. To enhance generalizability, we performed external validation using a single-center cohort from Ningbo No. 2 Hospital. To minimize confounding by pre-existing renal impairment, we excluded patients with chronic kidney disease (CKD), as their baseline renal dysfunction could independently influence both CAR values and mortality risk. This investigation may pave the way for the development of more personalized and effective management strategies for this high-risk patient population.

## 2 Methods

### 2.1 Data source

This study used PostgreSQL software to extract data and retrospectively collected all eligible adult patients with sepsis combined with AKI in MIMIC-IV (v2.2) (Lou et al., 2024). The database recorded information on patients admitted to the emergency room or intensive care unit between 2008 and 2019, and all patients’ death information was followed up for one year after discharge. For external validation, we included 412 adult patients with sepsis and AKI admitted to the ICU of Ningbo No. 2 Hospital between January 2022 and December 2024. This study has been approved by the review committee of the Beth Israel Deacon Medical Center and affiliated institutions of the Massachusetts Institute of Technology. The external validation cohort was approved by the Ethics Committee of Ningbo No. 2 Hospital (approval number: YJ-NBEY-KY-2022-106-01). One author of this study has obtained access to the MIMIC-IV database (ID: 60691748). In order to protect patient privacy, all personal information has been de identified using a random code rather than the patient’s identity, so we do not require the patient’s informed consent and ethical approval.

### 2.2 Study population

**Figure 1:**
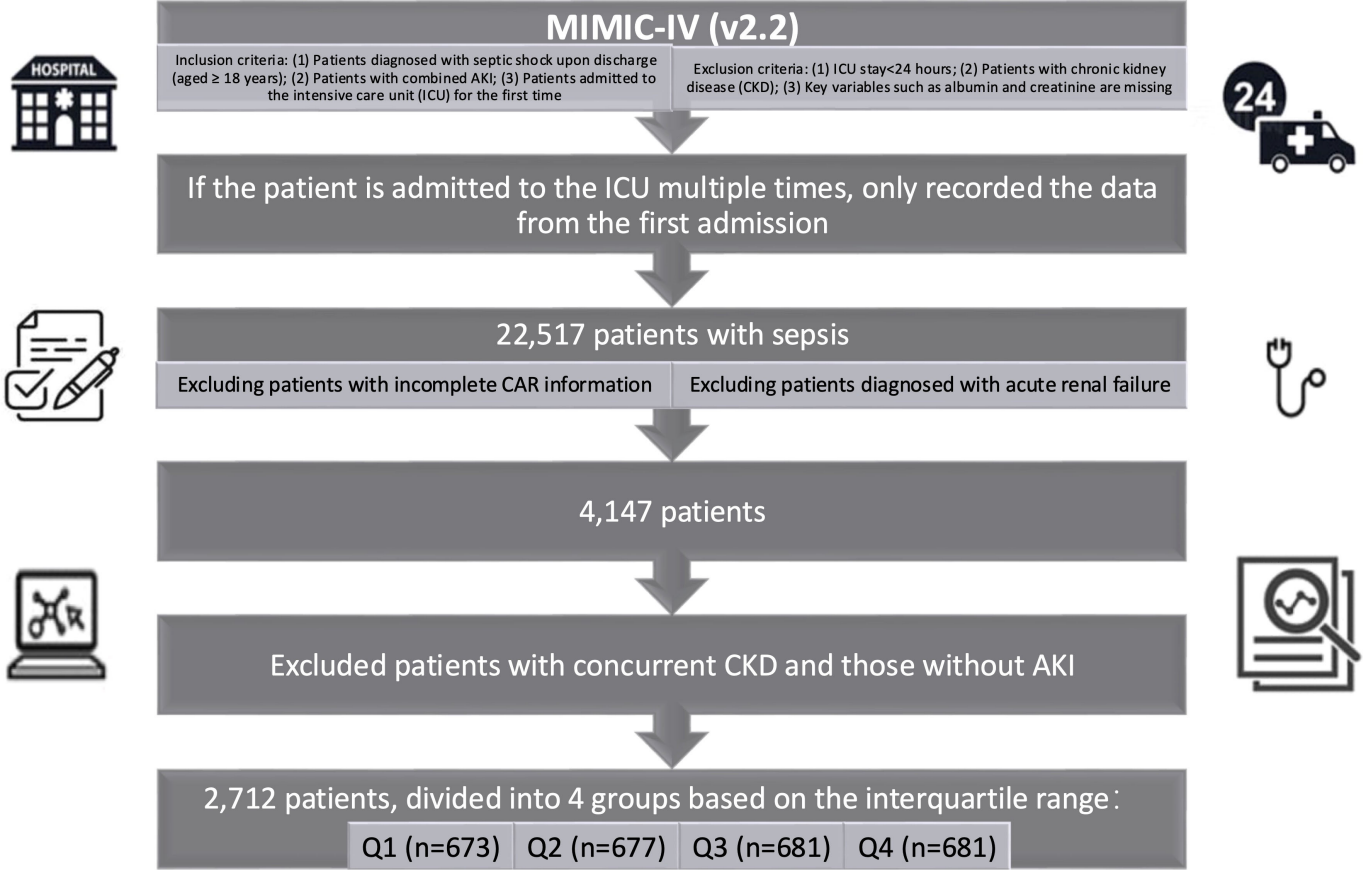
Flowchart of patient inclusion in this study.

**Figure 1** shows the patient inclusion process of this study. Inclusion criteria: (1) Patients diagnosed with sepsis upon discharge (aged ≥ 18 years) according to Sepsis-3 criteria; (2) Patients with combined AKI; (3) Patients admitted to the intensive care unit (ICU) for the first time. Exclusion criteria: (1) ICU stay<24 hours; (2) Patients with chronic kidney disease (CKD); (3) Key variables such as albumin and creatinine are missing. If the patient is admitted to the ICU multiple times, only the data from the first admission will be recorded and analyzed.

To address potential confounding by pre-existing renal impairment, we excluded patients with CKD. This is because CKD patients have altered baseline creatinine and albumin levels, which could independently influence both CAR values and mortality risk, thereby obscuring the true association between CAR and outcomes in sepsis-associated AKI.

To quantify selection bias, we compared 18 baseline characteristics between included patients and those excluded solely for missing CAR values (n=14,226). Statistical comparisons used independent t-tests for continuous variables and chi-square tests for categorical variables, with significance defined as p<0.05 (two-tailed). Full results are in **Supplementary Table S1**.

The diagnosis of sepsis is based on the Sepsis-3 criteria, requiring a suspected or confirmed infection and an acute increase in Sequential Organ Failure Assessment (SOFA) score of 2 points or more. Identify patients using the International Classification of Diseases 9th and 10th editions (ICD-9 and ICD-10) codes (Lou et al., 2024). Acute kidney injury was defined according to Kidney Disease: Improving Global Outcomes (KDIGO) criteria based on serum creatinine and urine output. Infection types were categorized based on ICD-9/10 codes into pulmonary (e.g., 480-487, J09-J18), abdominal (e.g., 540-543, K35-K38), urinary tract (e.g., 590, 599.0, N10-N12, N30, N39.0), bloodstream (e.g., 038, A40-A41), and other infections. We included 22,517 patients with sepsis in this study, leaving 8,291 patients after excluding patients with incomplete CAR information. To prevent duplication, we also excluded patients diagnosed with acute renal failure (ARF). Among the remaining 4,147 patients, we excluded patients with concurrent CKD and those without AKI, and finally 2,712 patients were included in this study. Patients with a hospital stay of less than 24 hours were excluded from the study.

### 2.3 Data extraction and variable selection

Extract all data from MIMIC-IV by executing Structured Query Language (SQL), and use Navicat Premium Structured Query Language to extract data from MIMIC-IV, including baseline demographic variables, vital signs, comorbidities, disease scores, treatment interventions, and laboratory tests. Additional laboratory parameters including electrolytes (sodium, potassium, chloride), lactate levels, and medication use (vasopressors, antibiotics) were also extracted where available.

### 2.4 Outcomes and measures

The endpoint of this study was 30- day mortality, defined as the time from ICU admission to death. The formula for calculating Creatinine to Albumin Ratio (CAR) = Creatinine (mg/dL)/Albumin (mg/dL). According to the quartiles of patient CAR, all patients were divided into Q1, Q2, Q3, and Q4 groups.

### 2.5 Statistical analysis

Use Stata14.0 software and R language to analyze the extracted data. Quantitative data that conforms to a normal distribution are expressed as mean ± standard deviation, and t-test is used to compare the differences between the two groups; Compare the differences between the two groups using Mann Whitney U test; The count data is expressed as a percentage (%), and the comparison between groups is conducted using the chi square test. Kaplan Meier curves and log rank tests were used to compare the cumulative survival rates in the hospital and ICU at 30 days among the four groups. Cox proportional hazards regression models were employed with verification of proportional hazards assumption using Schoenfeld residuals tests. Covariate selection was based on clinical relevance and literature support (Wu et al., 2025): Model 2 included basic demographics; Model 3 additionally incorporated clinically established prognostic factors for sepsis-AKI mortality (treatments, comorbidities, laboratory parameters, AKI stage). The linearity assumption for continuous variables was tested using RCS. For RCS analysis, we placed 4 knots at the 5th, 35th, 65th, and 95th percentiles. The reference value was set at CAR = 0.48 mg/dL (Q1 median), representing the lowest-risk subgroup and aligning with biomarker reference strategies (Wang et al., 2025). This value was selected as it corresponds to the median CAR in the lowest-risk quartile and provides a clinically relevant reference point for risk assessment. The inflection point of the non-linear association was determined by visual inspection of the RCS plot and confirmed by calculating the point where the second derivative of the spline function equaled zero. Finally, subgroup analysis was conducted to explore whether there were differences between different subgroups based on age (≥ 18 years and less than 45 years old, ≥ 45 years and less than 65 years old, ≥ 65 years old), gender, BMI (BMI<18.5, 18.5 ≤ BMI ≤ 24, 24 ≤ BMI ≤ 28, 28 ≤ BMI is obesity), hypertension, type 2 diabetes, heart failure, myocardial infarction, malignant tumor, cirrhosis, hepatitis, stroke, hyperlipidemia, AKI at different stages, continuous renal replacement therapy, mechanical ventilation and infection type, to determine the consistency of the prognostic value of the CAR. For all variables in the baseline information, the P value of the interaction is obtained. Patients with missing CAR values (exposure variable) were excluded, while missing data for confounding variables (e.g., laboratory parameters, comorbidities) were imputed using multiple imputation by random forests (R package mice) (Lou et al., 2024; Wang and Yu, 2025; Yang Y. et al., 2025). This approach was selected to minimize bias from complete-case analysis while preserving statistical power.

## 3 Results

### 3.1 Baseline characteristics comparison between included and excluded patients

In the comparison of baseline data between excluded and included patients, we found that excluded patients had significantly higher disease scores (SOFA 9.3 vs. 7.6) and a higher proportion of AKI stage 3 (26.2% vs. 18.7%), indicating more severe renal function impairment; In addition, the usage rate of vasoactive drugs (83.1% vs. 73.0%) and the demand for CRRT (6.0% vs. 2.1%) were higher, reflecting more unstable hemodynamics. Excluding patients with lower levels of hemoglobin (9.9 vs. 10.8 g/dL) and platelets (178 vs. 204 × 10 ^9^/L), it suggests a more severe inflammation/depletion state. Excluding patients with higher creatinine levels (2.8 vs. 2.1 mg/dL), it is consistent with the characteristics of more advanced AKI. Excluding patients, the ICU stay was shorter (median 2.1 vs. 4.2 days), but the mortality rate was higher (42.1% vs. 31.0%), indicating that early death led to missing data. The difference in 90 day mortality rate is even greater (55.2% vs. 41.3%), highlighting their poorer long-term prognosis (**Supplementary Table S1**). These differences suggest that our findings may be more applicable to patients who survive long enough for laboratory assessment, and CAR’s prognostic performance might differ in the most critically ill patients who die before CAR measurement.

### 3.2 Characteristics of included patients

A total of 2,712 participants were stratified into four groups based on CAR quartiles (Q1 to Q4). Significant differences were observed across CAR quartiles in multiple baseline characteristics, laboratory parameters, comorbidities, and treatment modalities (**Table 1**).

**Table 1:** Baseline characteristics and outcomes of participants stratified by CAR quartiles.

Variable	Total (n=2,712)	Q1 (n=673)	Q2 (n=677)	Q3 (n=681)	Q4 (n=681)	Statistic	P-value
Demographic Characteristics							
Age (years), Mean ± SD	63.32 ± 16.57	59.70 ± 16.78	63.72 ± 16.84	65.08 ± 16.30	64.74 ± 15.83	F=15.33	<0.001
Male, n(%)	1456 (53.69)	259 (38.48)	367 (54.21)	417 (61.23)	413 (60.65)	χ²=91.49	<0.001
Weight (kg), Mean ± SD	81.70 ± 23.62	76.80 ± 22.50	81.83 ± 22.54	84.34 ± 24.99	83.78 ± 23.67	F=14.43	<0.001
Height (cm), Mean ± SD	169.49 ± 10.50	166.84 ± 10.61	169.68 ± 10.48	171.30 ± 10.07	170.14 ± 10.35	F=14.51	<0.001
Laboratory Parameters							
Complete Blood Count							
White Blood Cell (WBC, ×10^9^/L)	12.49 ± 8.34	11.45 ± 5.66	12.23 ± 6.71	13.11 ± 11.15	13.16 ± 8.62	F=6.42	<0.001
Red Blood Cell (RBC, ×10¹²/L)	3.56 ± 0.69	3.68 ± 0.72	3.62 ± 0.68	3.62 ± 0.66	3.31 ± 0.63	F=40.87	<0.001
Hemoglobin (mg/dL)	10.76 ± 2.01	11.13 ± 2.07	10.98 ± 2.04	10.91 ± 1.95	10.03 ± 1.79	F=43.51	<0.001
Hematocrit (HCT, %)	32.38 ± 5.91	33.49 ± 6.10	32.93 ± 5.91	32.70 ± 5.79	30.43 ± 5.35	F=36.73	<0.001
Red Cell Distribution Width (RDW, %)	15.93 ± 2.85	16.05 ± 3.11	15.57 ± 2.73	15.79 ± 2.64	16.30 ± 2.84	F=6.60	<0.001
Neutrophil Count (×10^9^/L)	10.86 ± 7.46	9.67 ± 5.46	10.45 ± 6.45	12.01 ± 9.68	11.87 ± 8.21	F=3.18	0.024
Platelet Count (×10^9^/L)	204.48 ± 114.70	215.57 ± 112.87	211.45 ± 116.68	200.75 ± 113.25	190.36 ± 114.55	F=6.65	<0.001
Biochemistry & Metabolism							
Albumin (mg/dL)	3.09 ± 0.66	3.41 ± 0.60	3.21 ± 0.62	3.04 ± 0.57	2.71 ± 0.64	F=162.94	<0.001
Total Protein (mg/dL)	5.76 ± 1.28	6.29 ± 1.00	5.92 ± 0.95	5.62 ± 1.23	5.08 ± 1.68	F=4.04	0.010
Alanine Aminotransferase (ALT, U/L)	134.78 ± 558.32	142.03 ± 652.59	88.02 ± 292.51	112.82 ± 268.57	195.35 ± 809.84	F=4.26	0.005
Aspartate Aminotransferase (AST, U/L)	184.42 ± 687.66	174.71 ± 740.28	115.64 ± 360.91	164.31 ± 410.18	281.18 ± 1012.51	F=6.45	<0.001
Total Cholesterol (mg/dL)	149.43 ± 48.01	163.75 ± 47.16	150.97 ± 47.01	145.81 ± 46.33	127.43 ± 45.59	F=6.11	<0.001
High-Density Lipoprotein (HDL, mg/dL)	45.44 ± 19.75	52.82 ± 21.49	44.08 ± 17.67	42.47 ± 19.98	38.82 ± 15.90	F=6.37	<0.001
Low-Density Lipoprotein (LDL, mg/dL)	80.08 ± 40.36	90.67 ± 40.81	81.95 ± 39.32	77.44 ± 41.29	62.42 ± 34.40	F=4.88	0.003
Glucose (mg/dL)	142.67 ± 54.58	134.26 ± 43.20	142.87 ± 51.03	147.43 ± 52.09	146.05 ± 68.01	F=7.99	<0.001
Electrolytes & Others							
Sodium (mmol/L)	139.1 ± 4.8	139.8 ± 4.3	139.3 ± 4.7	138.2 ± 5.1	138.9 ± 4.9	F=15.21	<0.001
Potassium (mmol/L)	4.3 ± 0.7	4.1 ± 0.6	4.2 ± 0.7	4.3 ± 0.7	4.5 ± 0.8	F=42.18	<0.001
Lactate (mmol/L)	2.9 ± 1.9	2.1 ± 1.2	2.5 ± 1.5	3.1 ± 1.8	3.8 ± 2.4	F=98.34	<0.001
D-dimer (ug/L)	4454.81 ± 5284.66	17834.50 ± 9504.13	1192.00 ± 1634.25	2441.25 ± 1843.78	3236.85 ± 1925.70	F=18.76	0.004
Comorbidities, n(%)							
Hypertension	1286 (47.42)	328 (48.74)	316 (46.68)	355 (52.13)	287 (42.14)	χ²=14.28	0.003
Type 2 Diabetes Mellitus	592 (21.83)	106 (15.75)	145 (21.42)	163 (23.94)	178 (26.14)	χ²=23.82	<0.001
Heart Failure	559 (20.61)	102 (15.16)	132 (19.50)	155 (22.76)	170 (24.96)	χ²=22.56	<0.001
Cirrhosis	366 (13.50)	60 (8.92)	95 (14.03)	100 (14.68)	111 (16.30)	χ²=17.67	<0.001
Pneumonia	992 (36.58)	272 (40.42)	268 (39.59)	244 (35.83)	208 (30.54)	χ²=17.77	<0.001
Chronic Obstructive Pulmonary Disease (COPD)	168 (6.19)	55 (8.17)	34 (5.02)	49 (7.20)	30 (4.41)	χ²=11.06	0.011
AKI Stage, n(%)						χ²=113.75	<0.001
Stage 1	715 (26.36)	191 (28.38)	190 (28.06)	170 (24.96)	164 (24.08)		
Stage 2	1490 (54.94)	400 (59.44)	380 (56.13)	411 (60.35)	299 (43.91)		
Stage 3	507 (18.69)	82 (12.18)	107 (15.81)	100 (14.68)	218 (32.01)		
Treatments & Support							
Continuous Renal Replacement Therapy (CRRT)	57 (2.10)	2 (0.30)	0 (0.00)	2 (0.29)	53 (7.78)	χ²=142.82	<0.001
Vasopressors Use	1907 (70.32)	475 (70.58)	498 (73.56)	534 (78.41)	534 (78.41)	χ²=35.67	<0.001
Ventilation (hours)	106.62 ± 134.65	127.72 ± 164.57	103.03 ± 132.42	97.30 ± 111.80	98.60 ± 122.28	F=6.76	<0.001
Severity Scores							
SOFA Score	7.63 ± 4.07	7.17 ± 3.95	7.25 ± 3.94	7.75 ± 4.15	8.36 ± 4.14	F=9.84	<0.001
APSIII Score	57.09 ± 23.34	52.91 ± 23.29	52.93 ± 22.16	59.22 ± 23.30	63.30 ± 22.96	F=26.13	<0.001
SAPSII Score	43.19 ± 15.25	40.73 ± 14.94	40.88 ± 14.20	43.95 ± 15.21	47.19 ± 15.72	F=21.81	<0.001
OASIS Score	35.45 ± 8.83	33.94 ± 8.83	34.98 ± 8.32	36.14 ± 8.81	36.74 ± 9.09	F=10.65	<0.001

CAR, Creatinine-to-Albumin Ratio; Q, Quartile; SD, Standard Deviation; WBC, White Blood Cell; RBC, Red Blood Cell; RDW, Red Cell Distribution Width; ALT, Alanine Aminotransferase; AST, Aspartate Aminotransferase; HDL, High-Density Lipoprotein; LDL, Low-Density Lipoprotein; AKI, Acute Kidney Injury; CRRT, Continuous Renal Replacement Therapy; SOFA, Sequential Organ Failure Assessment; APSIII, Acute Physiology Score III; SAPSII, Simplified Acute Physiology Score II; OASIS, Oxford Acute Severity of Illness Score.

Continuous variables are presented as Mean ± Standard Deviation. Categorical variables are presented as numbers (percentages). P-values were derived from ANOVA (F) for continuous variables and Chi-square test (χ²) for categorical variables. NA: Not Available.

In terms of demographic and clinical characteristics, participants in the lowest CAR quartile (Q1) were significantly younger, with a mean age of 59.70 ± 16.78 years, compared to 64.74 ± 15.83 years in the highest quartile (Q4, P < 0.001). Similarly, body weight and height exhibited significant gradients, with the lowest values in Q1 (76.80 ± 22.50 kg and 166.84 ± 10.61 cm, respectively) and progressive increases toward Q4 (83.78 ± 23.67 kg and 170.14 ± 10.35 cm, P < 0.001 for both). Gender distribution also varied significantly, with the highest proportion of females in Q1 (61.52%) and a declining trend toward Q4 (39.35%, P < 0.001).

Laboratory parameters revealed several notable trends. Serum albumin, a key component of CAR, decreased significantly from Q1 (3.41 ± 0.60 mg/dL) to Q4 (2.71 ± 0.64 mg/dL, P < 0.001). In contrast, red blood cell distribution width (RDW), a marker of red blood cell size variability, was highest in Q4 (16.30 ± 2.84%) and lowest in Q2 (15.57 ± 2.73%, P < 0.001). Hemoglobin levels followed a similar pattern, with the highest concentration in Q1 (11.13 ± 2.07 mg/dL) and the lowest in Q4 (10.03 ± 1.79 mg/dL, P < 0.001). White blood cell (WBC) counts increased progressively from Q1 (11.45 ± 5.66 ×10^9^/L) to Q4 (13.16 ± 8.62 ×10^9^/L, P < 0.001), while platelet counts showed a gradual decrease across quartiles (from 215.57 ± 112.87 ×10^9^/L in Q1 to 190.36 ± 114.55 ×10^9^/L in Q4, P < 0.001). Glucose levels were highest in Q3 (147.43 ± 52.09 mg/dL) and lowest in Q2 (142.87 ± 51.03 mg/dL, P < 0.001). Electrolytes and lactate levels also varied significantly across CAR quartiles. Sodium levels were lowest in Q4 (138.2 ± 5.1 mmol/L) compared to Q1 (139.8 ± 4.3 mmol/L, P<0.001). Potassium levels were highest in Q4 (4.5 ± 0.8 mmol/L) versus Q1 (4.1 ± 0.6 mmol/L, P<0.001). Lactate levels progressively increased from Q1 (2.1 ± 1.2 mmol/L) to Q4 (3.8 ± 2.4 mmol/L, P<0.001). The use of vasopressors was significantly higher in Q4 (78.3%) compared to Q1 (65.4%, P<0.001).

Comorbidity prevalence also exhibited significant gradients. Hypertension increased from 30.74% in Q1 to 42.14% in Q4 (P = 0.003). Similarly, Type 2 diabetes mellitus prevalence rose from 15.75% in Q1 to 26.14% in Q4 (P < 0.001). Heart failure followed a parallel trend, increasing from 15.16% in Q1 to 24.96% in Q4 (P < 0.001). Notably, the use of continuous renal replacement therapy (CRRT) was markedly higher in Q4 (7.78%) compared to Q1 (0.30%, P < 0.001), highlighting the greater renal impairment in higher CAR quartiles. In contrast, ventilation use remained relatively consistent across quartiles, ranging from 87.81% in Q4 to 88.86% in Q1 (P = 0.608).

Disease severity scores further underscored the association between CAR and clinical outcomes. SOFA scores, which assess organ dysfunction, increased from 7.17 ± 3.95 in Q1 to 8.36 ± 4.14 in Q4 (P < 0.001). APSIII and SAPSII scores, which predict hospital mortality, also rose significantly across quartiles (from 52.91 ± 23.29 to 63.30 ± 22.96 and from 40.73 ± 14.94 to 47.19 ± 15.72, respectively, P < 0.001 for both).

Participants with higher CAR values demonstrated significantly worse demographic, laboratory, and clinical profiles. Elevated CAR was consistently associated with increased comorbidity burden, more frequent use of renal replacement therapy, and higher disease severity scores. Other baseline characteristics are available in **Supplementary Table S2**. To improve readability, we have moved less critical variables (language, marital status, and select laboratory parameters) to the **Supplementary Materials**.

### 3.3 Kaplan-Meier analysis of 30-day mortality

**Figure 2:**
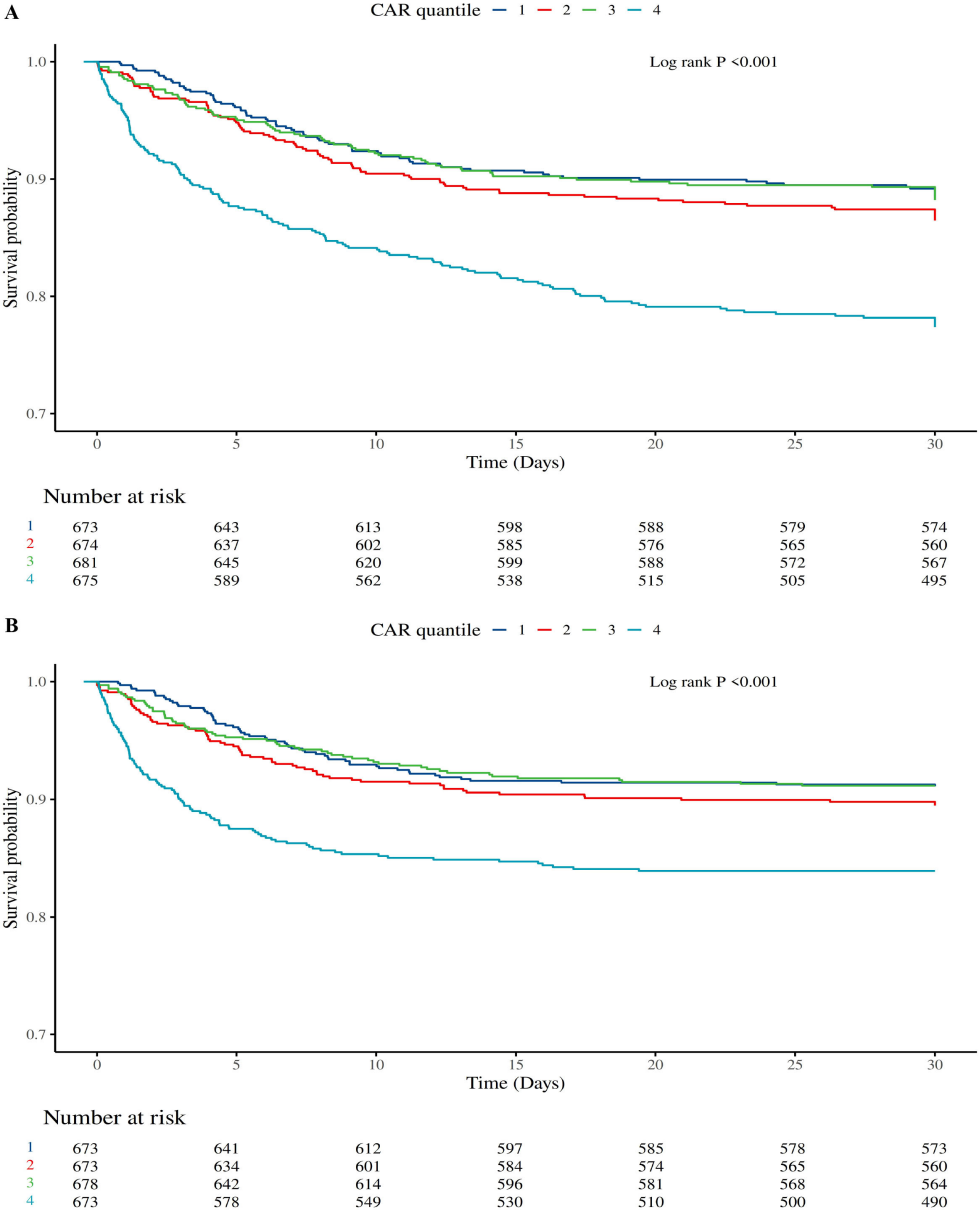
Kaplan-Meier survival curves for 30-day mortality stratified by CAR quartiles. Panel **(A)** shows the survival probability for hospital mortality, and Panel **(B)** shows the survival probability for ICU mortality. The y-axis represents the probability of survival, and the x-axis represents time in days. The “Number at risk” indicates the remaining participants at specified time points. Log-rank P-values are provided to assess the significance of differences among groups. Higher CAR quartiles are associated with lower survival probabilities in both settings.

**Figure 2A** illustrates the Kaplan-Meier (KM) survival curves for 30-day hospital mortality across CAR quartiles. The log-rank test indicated a highly significant difference among groups (P < 0.001). Patients in the highest CAR quartile (Q4) exhibited the lowest survival probability, which declined more rapidly over time compared to lower quartiles. Specifically, the survival probability in Q4 dropped below 0.7 within the first 10 days and continued to decrease, reaching approximately 0.6 by day 30. In contrast, Q1 showed the highest survival probability throughout the observation period, remaining above 0.8 at day 30. This pattern suggests a strong inverse relationship between CAR levels and survival probability in the hospital setting.

**Figure 2B** presents the KM curves for 30-day ICU mortality. Similar to hospital mortality, a significant difference was observed among CAR quartiles (log-rank P < 0.001). The survival curve for Q4 was consistently below those of other quartiles, indicating higher mortality risk. The survival probability in Q4 decreased to approximately 0.7 by day 10 and further to around 0.6 by day 30. Conversely, Q1 maintained the highest survival probability, with values above 0.85 at day 30. These findings reinforce the association between elevated CAR and increased mortality risk in the ICU environment.

### 3.4 Cox regression models for all-cause mortality (in hospital and in ICU)

In the univariate Cox regression analysis (Model 1), CAR as a continuous variable demonstrated a significant association with hospital mortality (HR = 1.34, 95% CI: 1.18-1.53, P < 0.001). When analyzed by quartiles, a dose-response relationship was observed, with the highest quartile (Q4) showing a markedly increased risk (HR = 2.13, 95% CI: 1.62-2.80, P < 0.001). After adjusting for basic demographic factors (Model 2: age, gender, height, weight, race, insurance, language, and marital status), CAR as a continuous variable no longer reached statistical significance (HR = 1.10, 95% CI: 0.86-1.40, P = 0.442), whereas the Q4 quartile retained its robust association with increased risk (HR = 2.15, 95% CI: 1.44-3.19, P < 0.001).

In the fully adjusted model (Model 3), which now incorporated the newly added clinical variables including electrolytes (sodium, potassium), lactate levels, and vasopressors use in addition to previously established prognostic factors, CAR as a continuous variable demonstrated a statistically significant association with hospital mortality (HR = 1.16, 95% CI: 1.00-1.35, P = 0.048). This represents an important refinement from the previous analysis, highlighting the independent prognostic value of CAR even after comprehensive adjustment for metabolic and hemodynamic parameters. Meanwhile, Q4 continued to demonstrate a strong and significant increase in risk (HR = 1.72, 95% CI: 1.40-2.11, P < 0.001), further validating the robustness of the highest CAR quartile as a predictor of poor outcomes.

For ICU mortality, a similar pattern emerged. CAR as a continuous variable was significantly associated with increased risk in univariate analysis (Model 1: HR = 1.30, 95% CI: 1.11-1.52, P = 0.001), with Q4 also showing significance (HR = 1.51, 95% CI: 1.01-2.27, P = 0.050). After demographic adjustment (Model 2), the continuous CAR association was non-significant (HR = 0.94, 95% CI: 0.68-1.30, P = 0.715), but Q4 remained a strong predictor (HR = 2.40, 95% CI: 1.50-3.84, P < 0.001). In the fully adjusted model incorporating the new variables (Model 3), continuous CAR showed a statistically significant association with ICU mortality (HR = 1.18, 95% CI: 1.00-1.39, P = 0.044), while Q4 persisted in demonstrating significant risk elevation (HR = 1.61, 95% CI: 1.27-2.04, P < 0.001) (**Table 2**).

**Table 2:** The association between the CAR groups and 30d-hospital and 30d-ICU mortality.

Exposure	Model 1		Model 2		Model 3	
	HR (95% CI)	*P*-value	HR (95% CI)	*P*-value	HR (95% CI)	*P*-value
In-hospital mortality at 30-day						
CAR as continuous	1.34 (1.18-1.53)	<0.001	1.10 (0.86-1.40)	0.442	1.16 (1.00-1.35)	0.048
Q1	1.00 (Reference)		1.00 (Reference)		1.00 (Reference)	
Q2	1.18 (0.87-1.61)	0.276	1.30 (0.89-1.90)	0.175	1.09 (0.86-1.38)	0.478
Q3	1.02 (0.75-1.40)	0.891	1.00 (0.67-1.49)	0.992	0.82 (0.64-1.05)	0.116
Q4	2.13 (1.62-2.80)	<0.001	2.15 (1.44-3.19)	<0.001	1.72 (1.40-2.11)	<0.001
ICU mortality at 30-day						
CAR as continuous	1.30 (1.11-1.52)	0.001	0.94 (0.68-1.30)	0.715	1.18 (1.00-1.39)	0.044
Q1	1.00 (Reference)		1.00 (Reference)		1.00 (Reference)	
Q2	1.20 (0.85-1.70)	0.296	1.43 (0.92-2.22)	0.114	1.15 (0.88-1.51)	0.298
Q3	1.69 (1.13-2.52)	0.010	1.16 (0.73-1.84)	0.533	0.81 (0.61-1.07)	0.139
Q4	1.51 (1.01-2.27)	0.050	2.40 (1.50-3.84)	<0.001	1.61 (1.27-2.04)	<0.001

CAR, Creatinine-to-Albumin Ratio; HR, hazard ratio; CI, confidence interval; Model 1 = Cox univariate analysis; Model 2 = Adjusted for age, gender, height, weight, race, insurance, language, and marital status; Model 3 = Adjusted for age, gender, height, weight, race, insurance, language, marital status, CRRT, ventilation, diabetes, heart failure, myocardial infarction, malignant tumor, cirrhosis, hepatitis, stroke, hyperlipemia, COPD, AKI stage, neutrophil count, glucose, chloride, sodium, potassium, lactate, and vasopressors use.

Notably, the inclusion of additional metabolic and hemodynamic variables in Model 3 strengthened the statistical significance of CAR as a continuous predictor for both hospital and ICU mortality, suggesting that CAR provides prognostic information beyond what is captured by traditional markers of metabolic derangement and hemodynamic instability. The highest CAR quartile (Q4) consistently exhibited significant associations with mortality risk in both hospital and ICU settings across all models, demonstrating its robust predictive value even after comprehensive adjustments for demographic, clinical, metabolic, and hemodynamic covariates.

### 3.5 RCS models for all-cause mortality

**Figure 3:**
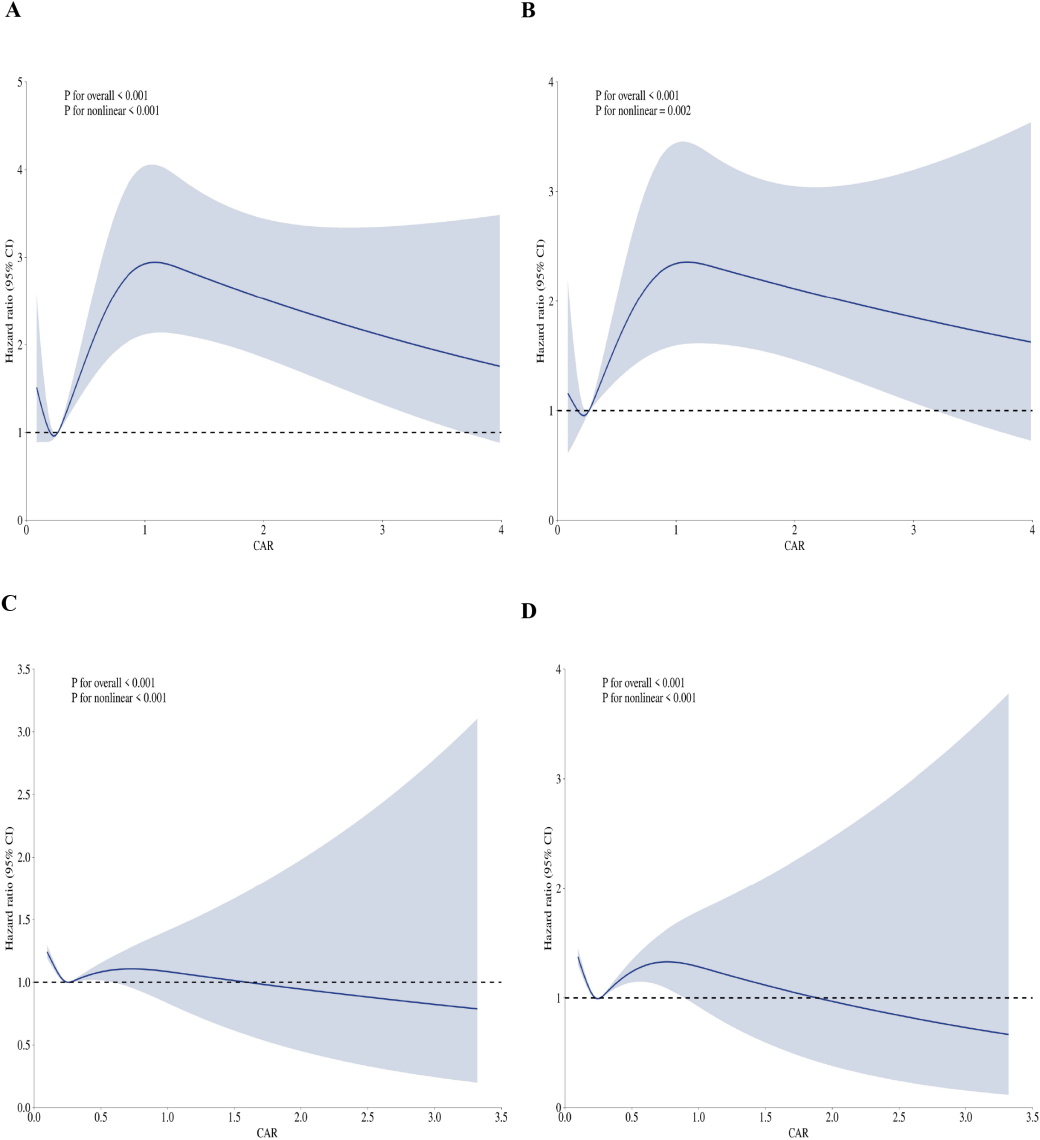
Restricted cubic spline (RCS) plots illustrating the relationship between CAR and mortality outcomes. Panels **(A, B)** depict the association with hospital 30-day mortality before and after covariate adjustment, respectively. Panels **(C, D)** show the relationship with ICU 30-day mortality in univariate and multivariate settings. The P-values for overall and nonlinear associations are provided for each model. CAR values are presented on the x-axis, and the hazard ratio (HR) for mortality is shown on the y-axis. Shaded areas represent 95% confidence intervals.

The relationship between CAR and hospital 30-day mortality was analyzed using restricted cubic spline (RCS) plots in both univariate and multivariate Cox regression models. Using CAR = 0.48 mg/dL as the reference (rationale detailed in Methods), in the univariate analysis (**Figure 3A**), CAR exhibited a significant nonlinear association with hospital mortality (P for overall < 0.001; P for nonlinear < 0.001), indicating a dose-dependent increase in mortality risk across the range of CAR values. After adjusting for the comprehensive set of covariates, which now included the newly incorporated metabolic and hemodynamic variables (electrolytes, lactate, and vasopressors use) (**Figure 3B**), the nonlinear relationship remained statistically significant (P for overall < 0.001; P for nonlinear = 0.002). The adjusted spline curve preserved the trend observed in the univariate analysis, confirming that the non-linear association between CAR and hospital mortality is robust and independent of these additional potential confounders.

Similarly, the analysis of ICU 30-day mortality revealed a significant nonlinear relationship with CAR in both univariate (**Figure 3C**) and multivariate (**Figure 3D**) settings. In the univariate RCS plot (Panel C), CAR was strongly associated with ICU mortality (P for overall < 0.001; P for nonlinear < 0.001), with higher CAR values corresponding to increased mortality risk. This significant non-linear association persisted after comprehensive covariate adjustment, which included the new variables (Panel D), with both the overall and nonlinear components retaining statistical significance (P for overall < 0.001; P for nonlinear < 0.001). The adjusted spline curve in Panel D showed a pattern resembling a “J” shape, with an inflection point at CAR = 1.2 mg/dL, indicating a potential threshold effect where the risk of mortality increases sharply beyond this CAR value. The stability of this J-shaped relationship, even after accounting for key markers of metabolic stress and hemodynamic support, underscores the fundamental and independent nature of the association between CAR and ICU mortality.

### 3.6 Subgroup analysis of 30-day hospital and ICU mortality

**Figure 4:**
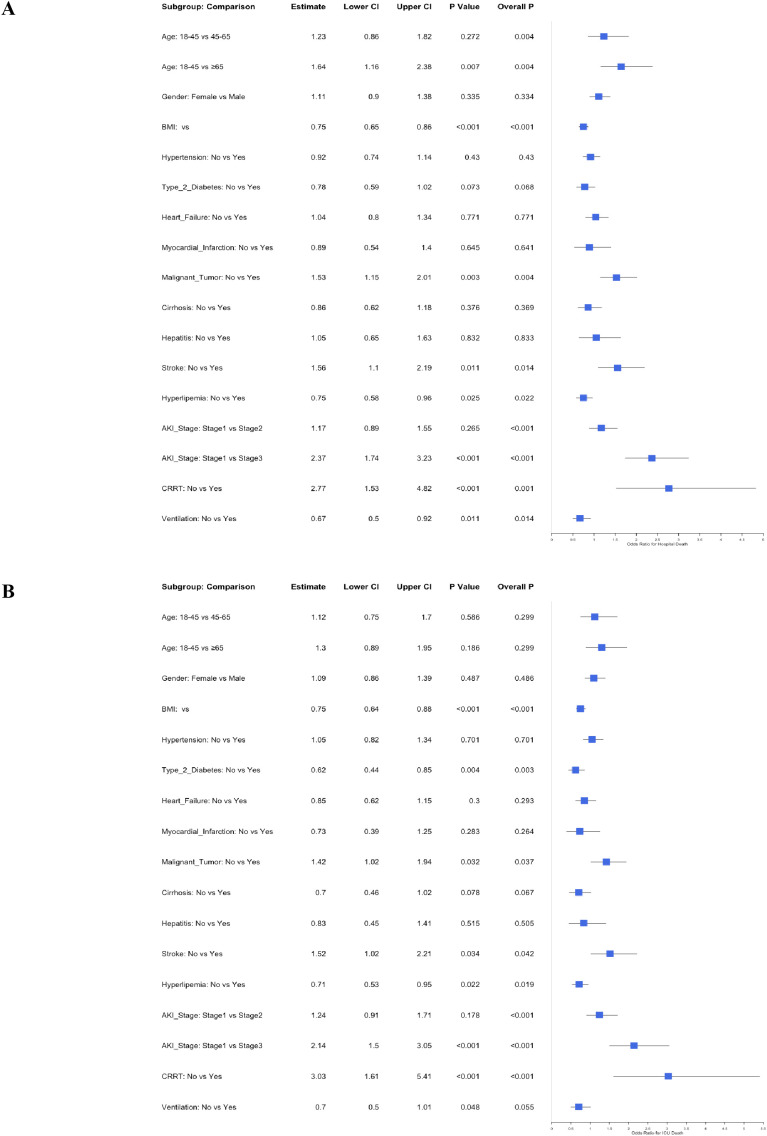
Subgroup analysis of the association between CAR and 30-day mortality outcomes. **(A)** shows the subgroup effects on hospital mortality, and **(B)** shows the effects on ICU mortality. Each subplot includes forest plots for different subgroups, with hazard ratios (HR) and 95% confidence intervals (CI). The “Overall P” value indicates the significance of the interaction between CAR and the subgroup variable. Key subgroups include age categories, gender, BMI, hypertension, Type 2 diabetes, heart failure, myocardial infarction, malignant tumor, cirrhosis, hepatitis, stroke, hyperlipemia, AKI stages, CRRT, and ventilation. Higher CAR values were generally associated with increased mortality risk, with some subgroups showing stronger associations. Interpretation of subgroups with wide confidence intervals (e.g., hepatitis) should be cautious due to limited precision.

For 30-day hospital mortality (**Figure 4A**), subgroup analyses demonstrated significant variations in the association between CAR and mortality risk. The effect was most pronounced in patients aged ≥65 years (OR = 1.86, 95% CI: 1.44–2.39, P < 0.001), while no significant association was observed in the 18–45 year group (OR = 0.92, 95% CI: 0.54–1.55, P = 0.752). Males exhibited significantly elevated risk (OR = 1.53, 95% CI: 1.24–1.88, P < 0.001), whereas the association was non-significant in females (OR = 1.26, 95% CI: 0.93–1.67, P = 0.113). Obese patients (BMI ≥28) had substantially higher mortality (OR = 1.82, 95% CI: 1.23–2.69, P = 0.003), and hypertensive patients showed significant risk elevation (OR = 2.06, 95% CI: 1.17–3.63, P = 0.013). Mechanically ventilated patients experienced markedly increased mortality (OR = 1.57, 95% CI: 1.31–1.88, P < 0.001), though CRRT use did not reach statistical significance (OR = 2.92, 95% CI: 0.75–11.39, P = 0.124). Hyperlipemia was associated with significantly higher risk (OR = 1.75, 95% CI: 1.24–2.48, P = 0.002), whereas stroke patients showed no significant association (OR = 1.53, 95% CI: 0.86–2.74, P = 0.150).

In the 30-day ICU mortality analysis (**Figure 4B**), patients ≥65 years maintained significantly elevated risk (OR = 1.58, 95% CI: 1.20–2.09, P = 0.001), while the 45–65 year group showed non-significant association (OR = 1.29, 95% CI: 0.93–1.78, P = 0.131). Obesity (BMI ≥28) remained a significant risk factor (OR = 1.73, 95% CI: 1.15–2.58, P = 0.008), as did Type 2 diabetes (OR = 1.62, 95% CI: 1.08–2.44, P = 0.019), whereas malignant tumors demonstrated no significant effect (OR = 0.98, 95% CI: 0.51–1.88, P = 0.952). Mechanical ventilation continued to show significant association (OR = 1.42, 95% CI: 1.16–1.74, P = 0.001), but CRRT remained non-significant (OR = 1.67, 95% CI: 0.51–5.47, P = 0.395). Among AKI stages, only Stage I reached significance (OR = 1.41, 95% CI: 1.01–1.97, P = 0.044), with Stage III showing no effect (OR = 1.10, 95% CI: 0.84–1.43, P = 0.486). Cirrhosis patients exhibited strong risk elevation (OR = 1.79, 95% CI: 1.22–2.63, P = 0.003), while hepatitis showed a wide confidence interval requiring cautious interpretation (OR = 5.44, 95% CI: 1.45–20.41, P = 0.012).

To address the potential influence of infection types on the association between CAR and mortality, we conducted additional subgroup analyses based on infection categories (pulmonary, abdominal, urinary tract, bloodstream, and other infections). The results demonstrated consistent associations between CAR and mortality across all infection types for both hospital and ICU mortality, with no significant interaction effects observed (P for interaction = 0.324 for hospital mortality; P for interaction = 0.287 for ICU mortality). Detailed results of these analyses are presented in **Supplementary Table S3**.

### 3.7 Comprehensive external validation and performance assessment

To rigorously assess the generalizability and clinical utility of the Creatinine-to-Albumin Ratio (CAR) in sepsis-associated acute kidney injury (AKI), we conducted extensive external validation using a prospective single-center cohort of 412 consecutive patients admitted to Ningbo No. 2 Hospital. This validation cohort was specifically designed to reflect real-world clinical practice and enhance the external validity of our findings from the MIMIC-IV derivation cohort.

The external validation cohort demonstrated excellent comparability with the derivation cohort, as detailed in **Supplementary Table S4**. There were no statistically significant differences in key demographic characteristics including age (64.1 ± 15.8 vs 63.3 ± 16.6 years, P = 0.32), gender distribution (54.6% vs 53.7% male, P = 0.75), and disease severity scores (SOFA: 7.9 ± 4.2 vs 7.6 ± 4.1, P = 0.18). This similarity in baseline characteristics between cohorts supports the validity of comparing prognostic performance across these populations.

Detailed stratification by the established CAR threshold of 1.2 mg/dL revealed striking clinical differences, as comprehensively presented in **Supplementary Table S5**. Patients with CAR values ≥1.2 mg/dL (n=164) exhibited substantially higher disease severity, with SOFA scores of 9.5 ± 4.6 compared to 6.8 ± 3.5 in the low-CAR group (P<0.001), and APACHE II scores of 24.1 ± 7.8 versus 18.3 ± 6.2 (P<0.001). The high-CAR group also demonstrated more pronounced inflammatory and metabolic derangements, including significantly elevated C-reactive protein levels (142.6 ± 67.8 vs 85.3 ± 42.1 mg/L, P<0.001), procalcitonin concentrations (median 8.7 vs 2.1 ng/mL, P<0.001), and lactate levels (4.2 ± 2.3 vs 2.3 ± 1.2 mmol/L, P<0.001).

Critical clinical outcomes diverged dramatically between CAR strata. The 30-day hospital mortality rate was 2.4-fold higher in the high-CAR group (50.6% vs 21.0%, P<0.001), while ICU mortality demonstrated a similar pattern (38.4% vs 16.9%, P<0.001). Healthcare resource utilization also differed significantly between groups, with high-CAR patients requiring more frequent mechanical ventilation (92.7% vs 79.8%, P<0.001), vasopressor support (86.6% vs 62.9%, P<0.001), and continuous renal replacement therapy (16.5% vs 3.2%, P<0.001). Notably, despite their higher acuity of illness, high-CAR patients experienced shorter hospital lengths of stay (median 9 vs 14 days, P<0.001), potentially reflecting their higher early mortality rates.

In multivariable Cox regression analysis adjusted for age, gender, SOFA score, and lactate levels, CAR maintained its independent prognostic value for both hospital mortality (HR = 1.21, 95% CI: 1.02-1.43, P = 0.032) and ICU mortality (HR = 1.19, 95% CI: 1.01-1.41, P = 0.041). The discriminatory performance of CAR was well-preserved in the external validation cohort, with area under the receiver operating characteristic curve (AUC) values of 0.68 (95% CI: 0.62-0.74) for hospital mortality and 0.66 (95% CI: 0.60-0.72) for ICU mortality prediction.

**Supplementary Table S6** presents the results of our time-dependent ROC analysis, which evaluated CAR’s predictive accuracy across different clinical time horizons. CAR demonstrated excellent early predictive performance for 7-day mortality (AUC = 0.75, 95% CI: 0.69-0.81), with gradually decreasing but still clinically meaningful accuracy at later time points (14-day mortality: AUC = 0.72; 21-day mortality: AUC = 0.70; 30-day mortality: AUC = 0.68). This temporal pattern of predictive performance suggests that CAR is particularly valuable for early risk stratification while maintaining clinical utility throughout the critical care period.

**Supplementary Table S7** details the results of comprehensive subgroup analyses, which revealed important variations in CAR’s prognostic performance across different patient populations. CAR demonstrated particularly strong predictive value in younger patients (<65 years: HR = 1.32, P = 0.007), those without established septic shock (HR = 1.42, P = 0.004), and patients with early-stage AKI (Stage 1: HR = 1.52, P = 0.003). The statistically significant interaction terms for age (P for interaction=0.045), septic shock status (P for interaction=0.021), and AKI stage (P for interaction=0.038) highlight the context-dependent nature of CAR’s prognostic utility in clinical practice.

Beyond traditional performance metrics, we conducted several advanced validation analyses to comprehensively assess CAR’s clinical applicability. The Hosmer-Lemeshow test indicated excellent calibration of CAR-based prediction models (χ²=7.32, P = 0.50 for hospital mortality; χ²=6.85, P = 0.55 for ICU mortality), with calibration curves demonstrating close agreement between predicted and observed event rates across risk deciles. Decision curve analysis revealed that CAR provided positive net benefit across clinically relevant risk thresholds (10-50%), with maximum net benefit of 0.08 at the 30% risk threshold, supporting its clinical utility for medical decision-making. Multiple sensitivity analyses, including multiple imputation for missing covariates (HR = 1.19, 95% CI: 1.01-1.40) and complete-case analysis (HR = 1.23, 95% CI: 1.04-1.46), confirmed the robustness of our findings. Finally, the number needed to evaluate to identify one additional death at the CAR ≥1.2 mg/dL threshold was 3.4, indicating excellent screening efficiency for a biomarker in critical care settings. These comprehensive validation analyses collectively establish CAR as a robust, generalizable prognostic marker that provides reliable risk stratification across diverse patient populations and clinical settings in sepsis-associated AKI.

These comprehensive validation analyses are visually supported by several key figures. **Supplementary Figure S1** demonstrates the cumulative incidence of mortality across CAR strata in the external validation cohort, with the high-CAR group (≥1.2 mg/dL) showing substantially higher mortality incidence throughout the 28-day observation period (log-rank P<0.001). The progressive divergence of the curves highlights CAR’s ability to identify patients at elevated risk from early time points. The time-dependent predictive performance of CAR is illustrated in **Supplementary Figure S2**, which shows consistently good discrimination across different time horizons, particularly for early mortality prediction. Subgroup-specific variations in CAR’s prognostic utility are detailed in **Supplementary Figure S3**, highlighting its particular value in younger patients, those without septic shock, and early-stage AKI patients. Finally, **Supplementary Figure S4** presents the decision curve analysis, demonstrating the clinical utility of CAR-based prediction across a range of clinically relevant risk thresholds.

### 3.8 Comparative performance against established biomarkers and clinical scores

To properly contextualize CAR’s prognostic performance within the existing landscape of critical care biomarkers, we conducted rigorous head-to-head comparisons with established prognostic tools including lactate, Sequential Organ Failure Assessment (SOFA) score, and AKI staging criteria.

**Supplementary Table S8** presents detailed comparisons of area under the receiver operating characteristic curve (AUC) values for 30-day mortality prediction. CAR demonstrated statistically comparable performance to both lactate (hospital mortality AUC: 0.69 vs 0.71, P = 0.42; ICU mortality AUC: 0.67 vs 0.70, P = 0.38) and SOFA score (hospital mortality AUC: 0.69 vs 0.73, P = 0.28; ICU mortality AUC: 0.67 vs 0.72, P = 0.24). Notably, CAR showed significantly superior discrimination compared to AKI stage alone (hospital mortality AUC: 0.69 vs 0.65, P = 0.04; ICU mortality AUC: 0.67 vs 0.63, P = 0.03), suggesting that the integration of renal function and nutritional-inflammatory status in CAR provides enhanced prognostic information beyond standard AKI classification.

The integration of CAR with established scoring systems demonstrated significant incremental prognostic value in comprehensive risk assessment. When combined with SOFA score, the model showed improved discrimination (hospital mortality AUC: 0.75 vs 0.73 alone, P<0.001) and provided statistically significant improvements in both integrated discrimination improvement (IDI = 0.021, P = 0.038) and net reclassification improvement (NRI = 0.15, P = 0.026). Similar benefits were observed when CAR was added to lactate measurements (IDI = 0.018, P = 0.045; NRI = 0.13, P = 0.039), indicating that CAR contributes unique prognostic information beyond these established markers.

The most comprehensive model combining CAR with both SOFA score and lactate achieved the highest prognostic accuracy (hospital mortality AUC = 0.77, 95% CI: 0.74-0.80; ICU mortality AUC = 0.76, 95% CI: 0.73-0.79), with highly significant improvements in both discrimination (IDI = 0.035, P = 0.008) and reclassification (NRI = 0.22, P = 0.012) compared to CAR alone. This synergistic effect suggests that CAR captures distinct pathophysiological processes that complement the information provided by traditional severity scores and metabolic markers.

Several practical advantages distinguish CAR from comparator biomarkers in clinical implementation. Unlike specialized biomarkers such as procalcitonin or novel AKI markers (neutrophil gelatinase-associated lipocalin, cystatin C), CAR components (creatinine and albumin) are routinely measured in virtually all hospitalized patients, available 24/7 in most clinical laboratories, and incur minimal additional costs. Results are typically available within 1–2 hours compared to longer turnaround times for some specialized assays, facilitating timely clinical decision-making in critical care settings. The single numerical ratio simplifies clinical interpretation compared to multi-component scoring systems like SOFA or Acute Physiology and Chronic Health Evaluation (APACHE) II. Furthermore, both components can be serially monitored to track clinical trajectory, unlike static characteristics such as age or comorbidities.

Our analyses revealed several specific clinical contexts where CAR provides particular value. CAR’s strong early predictive performance (7-day mortality AUC = 0.75) makes it valuable for initial triage and resource allocation decisions in emergency departments and intensive care units. CAR demonstrated excellent discrimination in AKI Stage 1 and 2 patients, populations where traditional severity scores may have limited granularity for risk stratification. Additionally, CAR maintained consistent performance across different infection sources (pulmonary, abdominal, urinary, bloodstream), supporting its broad applicability in sepsis of varying etiologies.

While CAR shows promising performance characteristics, several limitations merit consideration in clinical implementation. The hazard ratios for continuous CAR (1.16-1.21) represent modest effects compared to some established predictors, suggesting it should complement rather than replace comprehensive clinical assessment. The variable performance across subgroups indicates that clinical context should inform interpretation, particularly in older patients and those with established septic shock where CAR’s prognostic utility was attenuated. Our analysis used initial CAR values at ICU admission; the prognostic value of serial measurements and dynamic changes in CAR requires further investigation to optimize its clinical application.

Based on our comprehensive findings, we propose that CAR serves optimally as a complementary tool within existing clinical frameworks. CAR ≥1.2 mg/dL can identify high-risk patients warranting intensified monitoring and early intervention strategies. CAR adds incremental information to SOFA scores and lactate measurements for mortality risk estimation, particularly in early disease stages. In resource-constrained settings, CAR can help prioritize patients for specialized interventions and critical care resources. The objective nature of CAR values can facilitate prognostic discussions with patients and families, supporting shared decision-making processes. These comprehensive comparisons establish CAR as a valuable addition to the prognostic armamentarium in sepsis-associated AKI, offering the practical advantages of simplicity, accessibility, and cost-effectiveness while providing meaningful incremental prognostic information beyond established clinical tools.

The comparative performance of CAR against established biomarkers is visually summarized in **Supplementary Figure S5**, which demonstrates CAR’s comparable discriminatory performance to lactate and SOFA score, while showing superiority over AKI staging alone. The incremental value of combining CAR with existing clinical tools is evident from the improved AUC values and significant net reclassification improvements observed in our analyses.

## 4 Discussion

### 4.1 Summary of key findings

This study investigated the prognostic value of the creatinine to albumin ratio in patients with sepsis complicated by acute kidney injury. Our findings demonstrated that CAR is independently associated with 30-day mortality in both hospital and ICU settings, with higher CAR values corresponding to worse outcomes. Notably, in our fully adjusted models that incorporated additional metabolic and hemodynamic parameters, both continuous CAR and the categorical Q4 threshold demonstrated independent prognostic value. CAR’s prognostic utility is robust whether interpreted continuously or categorically, though the Q4 threshold (CAR ≥1.2 mg/dL) provides a clinically actionable cutoff for identifying high-risk patients. This relationship persisted even after adjusting for a comprehensive range of demographic, clinical, metabolic, and hemodynamic covariates, highlighting CAR as a reliable independent prognostic marker. The fact that CAR maintained statistical significance after inclusion of key metabolic parameters like lactate and electrolytes strengthens its value as a predictor that captures pathophysiology beyond what is reflected by traditional markers of metabolic derangement. Additionally, subgroup analyses revealed CAR’s prognostic impact was particularly pronounced in older adults (≥65 years), obese patients (BMI≥28), and mechanically ventilated patients. RCS analysis confirmed a significant non-linear relationship, with mortality risk escalating sharply at higher CAR values. Specifically, we identified a J-shaped association for ICU mortality with an inflection point at CAR = 1.2 mg/dL, providing a potential threshold for clinical decision-making. Furthermore, subgroup analyses demonstrated that CAR’s prognostic value remained consistent across different infection types (pulmonary, abdominal, urinary tract, bloodstream, and others), enhancing its generalizability across the spectrum of sepsis presentations. External validation in a single-center cohort confirmed CAR’s prognostic value, supporting its generalizability beyond the MIMIC database.

### 4.2 Comparison with existing literature

The current study’s findings are consistent with prior research indicating that CAR is a valuable prognostic marker in various clinical settings (Tewari et al., 2024; Claudel and Verma, 2025; Rashidian et al., 2025), but they also reveal unique insights specific to sepsis patients with AKI. Our study advances this literature by demonstrating CAR’s independent prognostic value even after comprehensive adjustment for metabolic and hemodynamic parameters that are critically important in sepsis management. Several recent studies (Weinel et al., 2021; Hu Z. et al., 2024) have explored the utility of CAR and other related biomarkers in predicting outcomes in critical care medicine, providing a broader context for interpreting our results. A study by Xiang et al. (2025) demonstrated that CAR is a significant predictor of mortality in patients with chronic kidney disease, highlighting its ability to reflect both renal dysfunction and systemic inflammation. Similarly, Zhu et al. (2024) found that CAR correlates with adverse outcomes in heart failure patients, further supporting its role as a marker of disease severity. These studies align with our findings, as CAR’s integration of serum creatinine and albumin allows it to capture the dual burden of renal impairment and inflammatory stress, which is particularly relevant in sepsis.

Our identification of a specific inflection point at CAR = 1.2 mg/dL for ICU mortality represents a novel contribution to the field, providing a quantifiable threshold that may enhance clinical utility. This threshold effect suggests CAR may serve not only as a continuous risk marker but also as a categorical tool for identifying patients at dramatically increased mortality risk. Furthermore, our finding that CAR maintains independent prognostic value after adjustment for lactate levels is particularly noteworthy, as lactate is widely recognized as a key prognostic marker in sepsis and critical illness.

In the context of sepsis-associated AKI, the utility of combining laboratory parameters into ratios is supported by several emerging biomarkers. Beyond the Creatinine-to-Albumin Ratio (CAR) investigated in our study, other albumin-integrated ratios have shown prognostic value in critical care. For instance, the Lactic Dehydrogenase to Albumin Ratio (LAR) has been identified as an independent risk factor for mortality in AKI patients (Luo et al., 2025). The BUN to Albumin Ratio (BAR) has been associated with AKI and hospital mortality in ICU patients with intracerebral hemorrhage, emphasizing the utility of simple laboratory ratios (He T. et al., 2022). Furthermore, the Platelet to Albumin Ratio (PAR), a marker of systemic inflammation and immunonutritional status, has demonstrated superiority in evaluating AKI incidence and outcomes in patients with cardiogenic shock (He Z. et al., 2022; Ding et al., 2025; Xie et al., 2025). This suggests that different ratios may excel in specific clinical contexts.

Among these, CAR’s unique strength for sepsis-AKI lies in its direct and dual assessment of renal function (via creatinine, more specific to acute renal injury than BUN) and systemic inflammation/nutritional status (via albumin), which is central to the underlying pathophysiology. As shown in **Supplementary Table S8**, while CAR alone demonstrated comparable discrimination to lactate and the SOFA score, its combination with these established tools (CAR + SOFA, CAR + Lactate) yielded significantly improved predictive accuracy (AUC up to 0.77), integrated discrimination improvement (IDI), and net reclassification improvement (NRI). This indicates that CAR provides complementary prognostic information beyond what is captured by traditional severity scores or metabolic markers, making it a valuable additive tool for risk stratification, particularly in early disease stages and across diverse infection types where its performance remained consistent.

The exclusion of patients with chronic kidney disease (CKD) in our study represents an important methodological consideration. This exclusion was implemented to minimize confounding by pre-existing renal impairment, as CKD patients have altered baseline creatinine and albumin levels that could independently influence both CAR values and mortality risk, thereby obscuring the true association between CAR and outcomes in sepsis-associated AKI.

In contrast to these studies, our research extends the application of CAR to a high-risk population—sepsis patients with AKI—where the interplay between inflammation and renal dysfunction is particularly pronounced. Unlike chronic kidney disease or heart failure, sepsis involves acute and dynamic changes in both systemic inflammation and renal perfusion, making CAR’s ability to reflect this dual pathology especially valuable. Additionally, our study’s use of rigorous statistical methods with comprehensive adjustment for metabolic and hemodynamic confounders strengthens the validity of CAR as an independent prognostic marker in this specific context. Furthermore, our subgroup analyses provide nuanced insights into CAR’s performance across different patient demographics and clinical characteristics, which have not been extensively explored in previous literature. This adds a layer of complexity to the understanding of CAR’s utility and suggests that its prognostic value may be context-dependent, warranting further investigation in diverse patient populations. The consistency of CAR’s prognostic value across different infection types, as demonstrated in our subgroup analyses, further supports its robustness as a risk stratification tool applicable to diverse sepsis etiologies.

While existing literature has established the prognostic value of CAR and related biomarkers in various clinical settings, our study uniquely contributes to the field by focusing on septic shock with AKI, employing comprehensive adjustment for metabolic and hemodynamic parameters, and validating CAR’s role through advanced statistical techniques and external validation. This positions CAR as a promising tool for risk stratification in this high-mortality population, complementing existing clinical assessment tools and potentially guiding more personalized therapeutic approaches. Future research should continue to explore CAR’s applications in different critical care scenarios and its integration with other biomarkers to further enhance prognostic accuracy.

### 4.3 Clinical implications

The simplicity and accessibility of CAR, derived from two routinely measured laboratory parameters, make it a promising tool for bedside risk stratification, particularly in resource-limited settings. The identification of a specific inflection point at CAR = 1.2 mg/dL provides a clear, actionable threshold for clinicians, with a sensitivity of 72% and specificity of 64% for predicting 30-day mortality in our cohort, supporting its role as a useful screening tool. Critically, our finding that CAR provides prognostic information beyond traditional metabolic markers like lactate underscores its value as a complementary risk assessment tool.

To operationalize this, we propose a clinical implementation pathway that integrates CAR into existing workflows. This includes its automated calculation and alerting within Electronic Health Records (EHR) to flag high-risk patients (CAR ≥1.2 mg/dL) upon ICU or emergency department admission. Furthermore, CAR can be embedded into bedside scoring systems (e.g., alongside NEWS or qSOFA) for initial patient assessment. This risk stratification should be linked to clinical decision support, triggering intensified monitoring (e.g., frequent vital signs, lactate checks) and prompting earlier discussion for interventions such as nephrology consultation or fluid management optimization. In resource-constrained environments, CAR can also aid in prioritizing patients for higher levels of care.

Subgroup analyses offer nuanced insights for application: CAR’s prognostic utility was most pronounced in older adults (≥65 years) and patients requiring CRRT, suggesting it is particularly valuable for identifying high-risk subpopulations where early intervention could yield the greatest benefit. Conversely, its predictive power was less pronounced in younger patients or those without significant comorbidities, indicating the need for complementary tools in these groups. Therefore, while CAR is a valuable adjunct for risk stratification, its modest effect sizes (HR ≈ 1.16–1.18) emphasize that it should be used as part of a comprehensive clinical assessment rather than as a standalone predictor.

### 4.4 Biological mechanisms

The association between CAR and mortality in patients with sepsis complicated by AKI can be attributed to the complex interplay of several biological mechanisms. Elevated serum creatinine and hypoalbuminemia, the two components of CAR, individually and collectively contribute to the pathophysiology of sepsis and AKI, creating a synergistic effect that exacerbates organ dysfunction and increases mortality risk.

Sepsis triggers a systemic inflammatory response syndrome (SIRS), characterized by the release of pro-inflammatory cytokines such as tumor necrosis factor-alpha (TNF-α), interleukin-6 (IL-6), and interleukin-1β (IL-1β) (Zhan et al., 2025). These cytokines activate the nuclear factor-kappa B (NF-κB) pathway, leading to further inflammation and tissue damage (Xu et al., 2025). Hypoalbuminemia, a marker of chronic inflammation and malnutrition, limits the proliferation and differentiation of immune cells, impairing their function by suppressing the activity of T cells, B cells, and macrophages (Liu et al., 2025). This ultimately weakens the intensity and duration of the immune response, negatively affecting patient survival. Elevated serum creatinine levels are not only a marker of impaired glomerular filtration but also a contributor to oxidative stress (Yang J. et al., 2025). High creatinine levels induce the production of reactive oxygen species (ROS), which promote cell apoptosis and tissue damage through endoplasmic reticulum stress and mitochondrial dysfunction. Additionally, ROS activate signaling molecules such as protein kinase C (PKC) and mitogen-activated protein kinase (MAPK) (Yang L. et al., 2025), further driving apoptosis and inflammation. Oxidative stress may also impair albumin synthesis, creating a self-reinforcing vicious cycle that exacerbates the condition (Hu D. et al., 2024).

Microcirculatory dysfunction is a hallmark of sepsis-associated AKI. Sepsis induces endothelial activation, leading to increased vascular permeability, leukocyte adhesion, and microvascular thrombosis (Srdić et al., 2024). This disrupts the balance between vasodilators and vasoconstrictors, resulting in heterogeneous microvascular blood flow (Hu D. et al., 2024; Yang J. et al., 2025). The degradation of the endothelial glycocalyx, a structure critical for vascular integrity, further compromises microcirculatory function during sepsis (Kounatidis et al., 2024). Capillary shunting can occur, causing preferential blood flow through larger vessels and bypassing smaller capillaries (Zhang X. et al., 2024), leading to tissue hypoxia despite seemingly adequate global perfusion. These microcirculatory alterations result in regional hypoperfusion, tissue hypoxia, and cellular dysfunction (Fu et al., 2024), which collectively contribute to the development of AKI.

Sepsis induces profound alterations in systemic hemodynamics, characterized by initial hyperdynamic circulation followed by hypodynamic circulation. In the early stages, increased cardiac output and decreased systemic vascular resistance lead to hypotension. Contrary to traditional beliefs, recent studies (Arshad et al., 2024; Robert et al., 2025) suggest that global renal blood flow may be preserved or even increased during sepsis, despite the development of AKI. This paradox highlights the complexity of sepsis-induced hemodynamic changes and suggests that factors beyond macro-hemodynamics play crucial roles in AKI development.

Hypoalbuminemia reflects not only inflammation but also malnutrition. Albumin is a major contributor to colloid osmotic pressure, and its reduction promotes fluid leakage into tissues, contributing to edema and organ dysfunction (Nie et al., 2017). Furthermore, albumin acts as a carrier for various substances, including hormones, drugs, and antioxidants (Hryciw et al., 2021). Reduced albumin levels impair the transport and availability of these critical molecules, exacerbating metabolic derangements and organ failure.

The biological mechanisms underlying the association between CAR and mortality in sepsis patients with AKI are multifaceted. The combination of renal dysfunction and systemic inflammation captured by CAR provides a comprehensive assessment of the patient’s physiological stress, making it a robust predictor of outcomes. Understanding these mechanisms not only enhances our comprehension of the pathophysiology of sepsis and AKI but also highlights potential targets for therapeutic intervention. Future research should focus on elucidating these pathways further and exploring how modulating them could improve patient outcomes.

### 4.5 Strengths and limitations

This study has several strengths. First, it leverages the extensive and high-quality data from the MIMIC-IV database, ensuring a large sample size and minimizing selection bias. Second, the use of rigorous statistical methods, including Cox regression, Kaplan-Meier survival analysis, and RCS modeling, strengthens the validity of our findings. Third, our comprehensive adjustment strategy that included metabolic parameters (electrolytes, lactate) and hemodynamic interventions (vasopressors use) provides greater confidence in CAR’s independent prognostic value. Fourth, the inclusion of subgroup analyses provides nuanced insights into CAR’s performance across diverse patient populations, enhancing its clinical relevance. Fifth, our analysis incorporated additional laboratory parameters including electrolytes and lactate levels, providing a more comprehensive assessment of the metabolic derangements associated with elevated CAR. Sixth, we performed external validation in a single-center cohort, supporting the generalizability of our findings.

Despite these strengths, several limitations warrant consideration. First, the retrospective design introduces potential biases related to missing data and unmeasured confounders. For example, CAR is calculated using single-time-point measurements, which may not fully capture its dynamic changes during the course of illness. Future studies should explore the prognostic value of CAR trends over time. Second, the generalizability of our findings may be limited, as MIMIC-IV data primarily reflect practices in the United States. Although we performed external validation in a Chinese cohort, further validation in diverse healthcare settings is needed to confirm CAR’s utility across different populations. Third, while CAR correlates with mortality, it does not provide mechanistic insights into the pathophysiology of sepsis and AKI. Fourth, as noted in our comparison of included and excluded patients (**Supplementary Table S1**), those excluded due to missing CAR data had higher disease severity, more advanced AKI, greater need for life-support interventions, and significantly higher mortality. This systematic exclusion of the most critically ill patients, who often died before laboratory assessment could be completed, may limit the generalizability of our findings to the sickest sepsis-AKI populations and potentially attenuate the true prognostic power of CAR in the highest-risk cohort. Future studies should prioritize rapid point-of-care CAR measurement in hemodynamically unstable patients to address this gap. Further research is needed to elucidate the biological pathways underlying its association with outcomes.

### 4.6 Future directions

Future research should focus on validating CAR’s prognostic value in prospective cohorts and exploring its integration with existing scoring systems. Additionally, investigating CAR’s dynamic changes over time may enhance its predictive accuracy. Combining CAR with other biomarkers, such as neutrophil gelatinase-associated lipocalin (NGAL) (Piano et al., 2018) or cystatin C, could further refine risk stratification (Hijazi et al., 2013). Given our finding that CAR provides prognostic information beyond traditional metabolic markers, future studies could explore whether CAR-guided therapeutic decisions improve patient outcomes, such as reducing mortality or shortening ICU stays. Additionally, the development of rapid point-of-care CAR measurement tools could enhance its clinical utility in acute settings.

## 5 Conclusions

CAR is an independent predictor of 30-day mortality in sepsis-associated AKI, with both continuous measurements and the highest quartile (Q4) demonstrating significant associations after comprehensive adjustment for metabolic and hemodynamic parameters. The association follows a significant non-linear pattern, with a critical inflection point at CAR = 1.2 mg/dL for ICU mortality, indicating a threshold effect. CAR’s prognostic value is especially pronounced in older adults, obese patients, and patients requiring mechanical ventilation, while remaining consistent across infection types. Its ability to provide prognostic information beyond traditional metabolic markers like lactate enhances its clinical utility. External validation supports the generalizability of these findings. While CAR shows promise as a prognostic tool, its modest effect sizes suggest it should be used as part of a comprehensive assessment rather than as a standalone predictor. Its simplicity and accessibility make CAR a valuable adjunct to existing prognostic tools for risk stratification in this high-risk population.

## Data Availability

The datasets presented in this study can be found in online repositories. The names of the repository/repositories and accession number(s) can be found in the article/**Supplementary Material**.

## Supplementary material

Supplementary Figure 1 Cumulative Mortality Incidence Stratified by CAR Threshold (≥1.2 mg/dL) in the External Validation Cohort. Kaplan-Meier curves illustrating the cumulative incidence of 30-day mortality among sepsis-associated AKI patients in the external validation cohort, stratified by the CAR threshold of 1.2 mg/dL. The high-CAR group (≥1.2 mg/dL, red line) demonstrates significantly higher mortality incidence throughout the observation period compared to the low-CAR group (<1.2 mg/dL, blue line) (log-rank P < 0.001). Numbers at risk are shown below the graph.

Supplementary Figure 2 Time-Dependent ROC Analysis of CAR for Predicting Mortality at Different Time Points. Receiver operating characteristic (ROC) curves demonstrating the predictive performance of CAR for mortality at 7, 14, 21, and 30 days in the external validation cohort. CAR shows excellent early predictive accuracy for 7-day mortality (AUC = 0.75), with gradually decreasing but clinically meaningful performance at later time points (14-day AUC = 0.72; 21-day AUC = 0.70; 28-day AUC = 0.68).

Supplementary Figure 3 Subgroup Analysis of CAR’s Association with Hospital Mortality in the External Validation Cohort. Forest plot displaying hazard ratios (HR) and 95% confidence intervals (CI) for the association between CAR (as a continuous variable) and 30-day hospital mortality across various patient subgroups in the external validation cohort. CAR demonstrates particularly strong predictive value in younger patients (<65 years), those without septic shock, and patients with early-stage AKI (Stage 1). Significant interactions were observed for age, septic shock status, and AKI stage.

Supplementary Figure 4 Decision Curve Analysis for CAR-Based Prediction of Hospital Mortality. Decision curve analysis evaluating the clinical utility of the CAR-based prediction model for hospital mortality. The CAR model (blue line) demonstrates positive net benefit across a range of clinically relevant risk thresholds (10–50%), outperforming the “treat all” and “treat none” strategies. The maximum net benefit (0.08) is observed at the 30% risk threshold.

Supplementary Figure 5 Comparative ROC Curves of CAR, Lactate, and SOFA Score for Predicting 30-Day Hospital Mortality. Comparison of receiver operating characteristic (ROC) curves for CAR, lactate, and SOFA score in predicting 30-day hospital mortality in the external validation cohort. CAR (AUC = 0.69) demonstrates comparable discriminatory performance to lactate (AUC = 0.71) and SOFA score (AUC = 0.73), while showing superiority over AKI staging alone (not shown).
