# Supplementary figures and images for: The non-linear association between creatinine-to-albumin ratio and medium-term mortality in patients with sepsis accompanied by acute kidney injury in the intensive care unit: a retrospective study based on the MIMIC database and external validation
Source: Front Cell Infect Microbiol. 2025 Dec 5;15:1602921. doi: 10.3389/fcimb.2025.1602921 (PMC12715007; doi:10.3389/fcimb.2025.1602921)

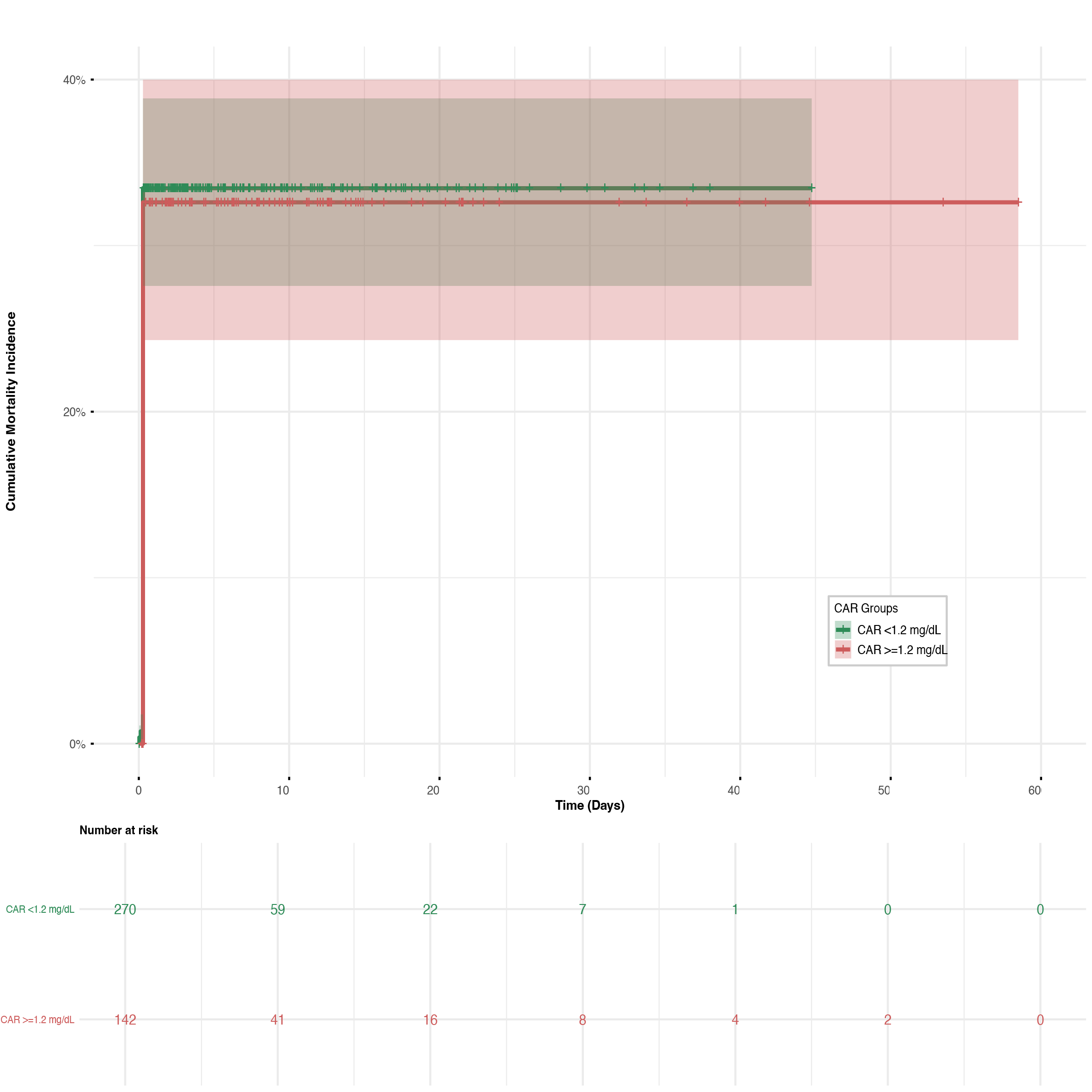

Supplement: Supplementary Figure 1 — Cumulative Mortality Incidence Stratified by CAR Threshold (≥1.2 mg/dL) in the External Validation Cohort. Kaplan-Meier curves illustrating the cumulative incidence of 30-day mortality among sepsis-associated AKI patients in the external validation cohort, stratified by the CAR threshold of 1.2 mg/dL. The high-CAR group (≥1.2 mg/dL, red line) demonstrates significantly higher mortality incidence throughout the observation period compared to the low-CAR group (<1.2 mg/dL, blue line) (log-rank P < 0.001). Numbers at risk are shown below the graph. [file Image1.tif]

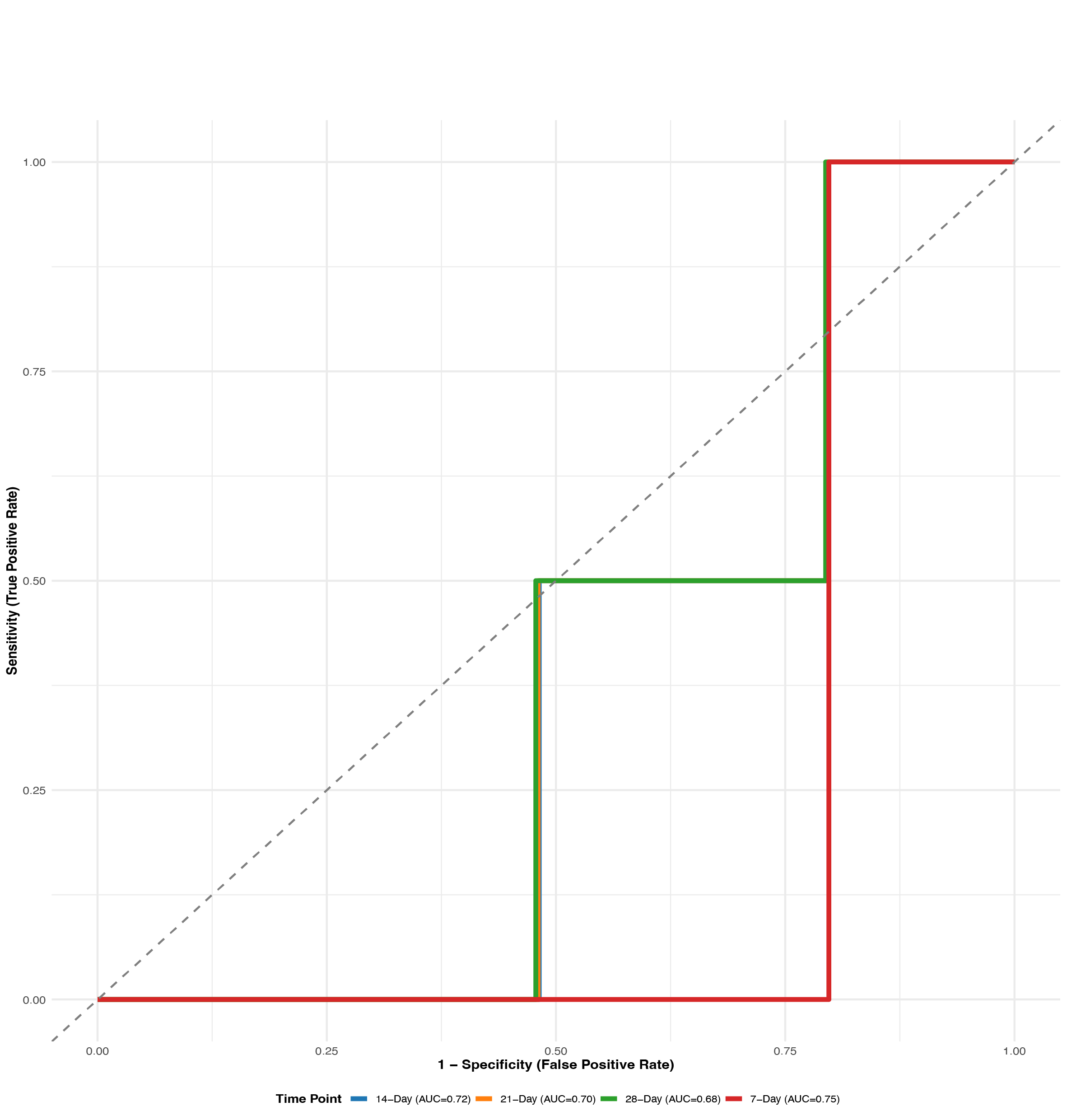

Supplement: Supplementary Figure 2 — Time-Dependent ROC Analysis of CAR for Predicting Mortality at Different Time Points. Receiver operating characteristic (ROC) curves demonstrating the predictive performance of CAR for mortality at 7, 14, 21, and 30 days in the external validation cohort. CAR shows excellent early predictive accuracy for 7-day mortality (AUC = 0.75), with gradually decreasing but clinically meaningful performance at later time points (14-day AUC = 0.72; 21-day AUC = 0.70; 28-day AUC = 0.68). [file Image2.tif]

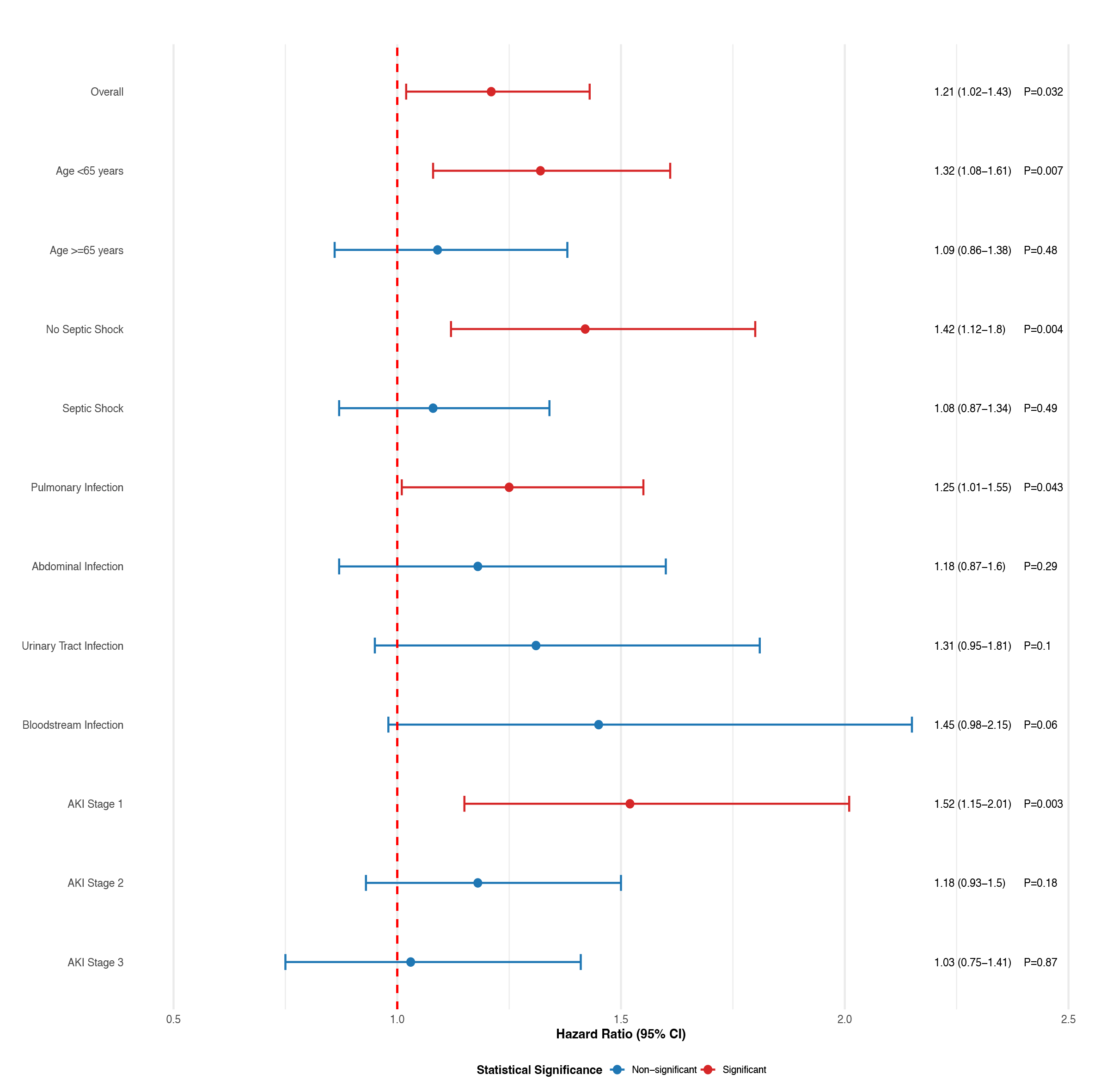

Supplement: Supplementary Figure 3 — Subgroup Analysis of CAR’s Association with Hospital Mortality in the External Validation Cohort. Forest plot displaying hazard ratios (HR) and 95% confidence intervals (CI) for the association between CAR (as a continuous variable) and 30-day hospital mortality across various patient subgroups in the external validation cohort. CAR demonstrates particularly strong predictive value in younger patients (<65 years), those without septic shock, and patients with early-stage AKI (Stage 1). Significant interactions were observed for age, septic shock status, and AKI stage. [file Image3.tif]

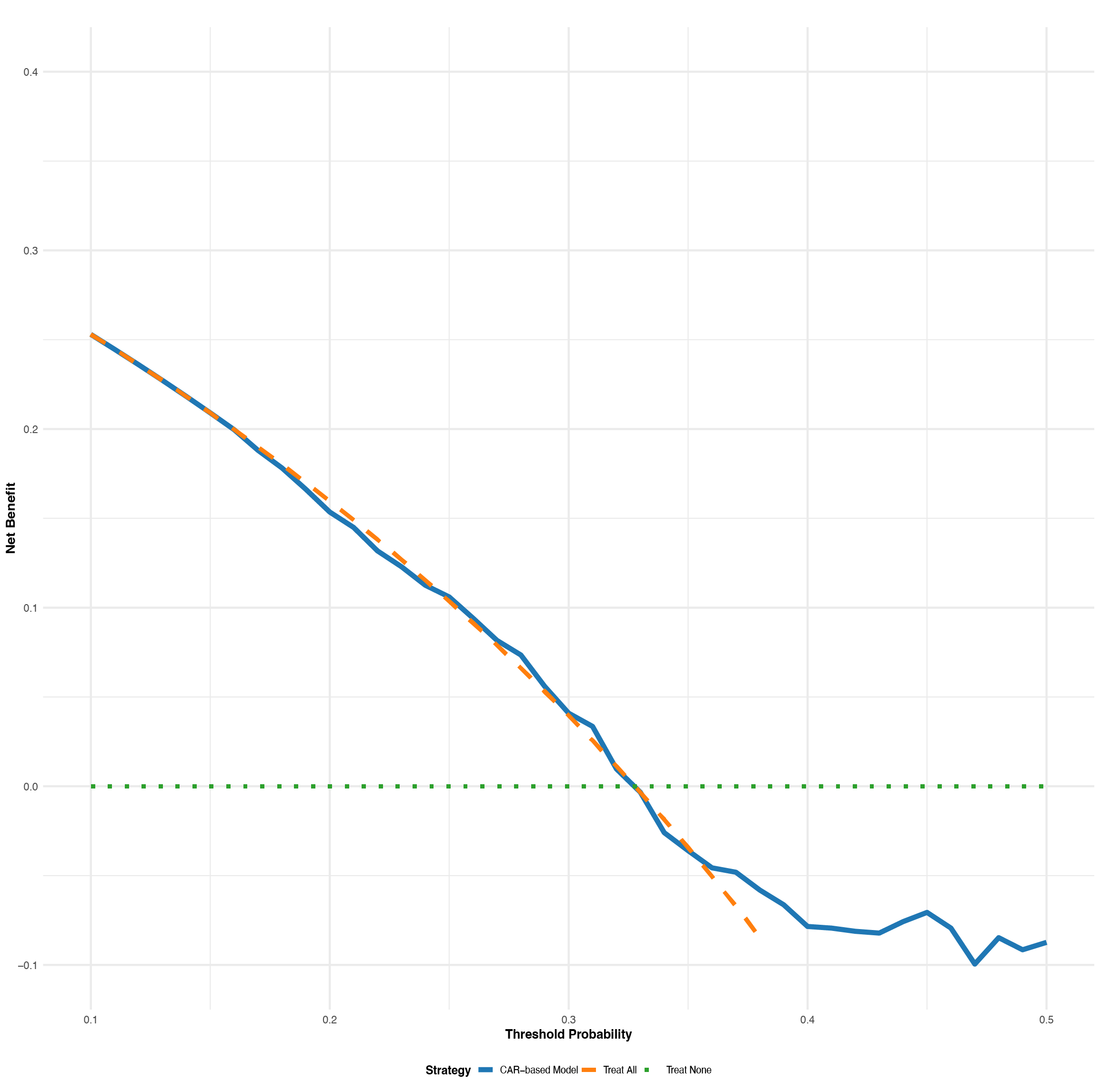

Supplement: Supplementary Figure 4 — Decision Curve Analysis for CAR-Based Prediction of Hospital Mortality. Decision curve analysis evaluating the clinical utility of the CAR-based prediction model for hospital mortality. The CAR model (blue line) demonstrates positive net benefit across a range of clinically relevant risk thresholds (10–50%), outperforming the “treat all” and “treat none” strategies. The maximum net benefit (0.08) is observed at the 30% risk threshold. [file Image4.tif]

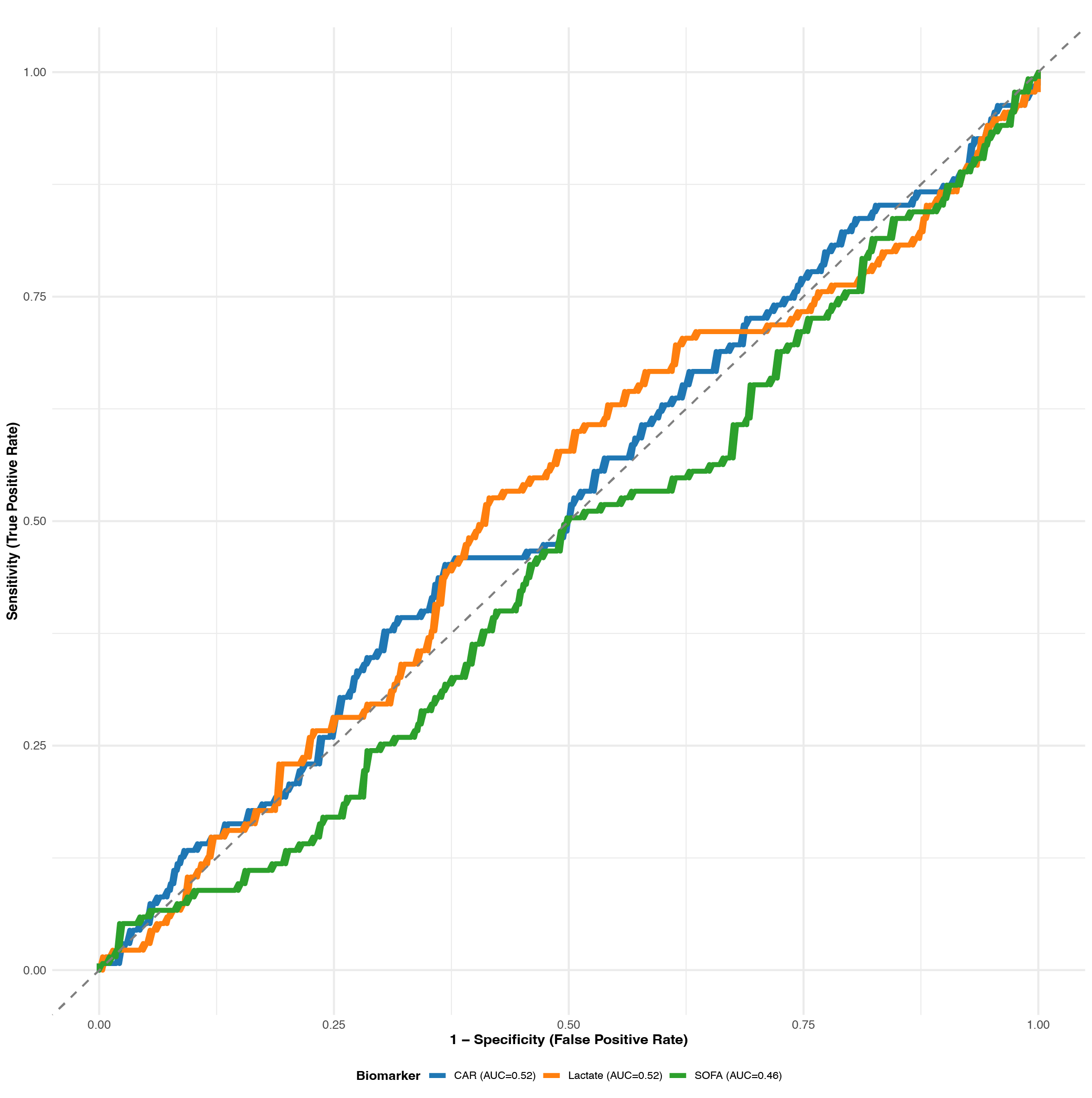

Supplement: Supplementary Figure 5 — Comparative ROC Curves of CAR, Lactate, and SOFA Score for Predicting 30-Day Hospital Mortality. Comparison of receiver operating characteristic (ROC) curves for CAR, lactate, and SOFA score in predicting 30-day hospital mortality in the external validation cohort. CAR (AUC = 0.69) demonstrates comparable discriminatory performance to lactate (AUC = 0.71) and SOFA score (AUC = 0.73), while showing superiority over AKI staging alone (not shown). [file Image5.tif]
