# Supplementary material for: The non-linear association between creatinine-to-albumin ratio and medium-term mortality in patients with sepsis accompanied by acute kidney injury in the intensive care unit: a retrospective study based on the MIMIC database and external validation
Source: Front Cell Infect Microbiol. 2025 Dec 5;15:1602921. doi: 10.3389/fcimb.2025.1602921 (PMC12715007; doi:10.3389/fcimb.2025.1602921)
Supplement: Supplementary file 6 [file Table1.docx]

| Supplementary Table S1. Baseline Characteristics Comparison Between Included and Excluded Patients | | | |
| --- | --- | --- | --- |
| Characteristic | Included Patients (n=2,712) | Excluded Patients (n=14,226) | P-value |
| ***Demographics*** |  |  |  |
| Age (years), Mean ± SD | 63.3 ± 16.6 | 67.8 ± 18.2 | <0.001 |
| Male Sex, n (%) | 1,456 (53.7%) | 7,920 (55.7%) | 0.08 |
| BMI (kg/m²), Mean ± SD | 28.1 ± 6.8 | 26.9 ± 7.2 | <0.001 |
| ***Disease Severity*** |  |  |  |
| SOFA Score, Mean ± SD | 7.6 ± 4.1 | 9.3 ± 4.7 | <0.001 |
| APSIII Score, Mean ± SD | 57.1 ± 23.3 | 65.4 ± 25.1 | <0.001 |
| SAPSII Score, Mean ± SD | 43.2 ± 15.3 | 50.1 ± 16.9 | <0.001 |
| ***Comorbidities, n (%)*** |  |  |  |
| Hypertension | 1,286 (47.4%) | 6,950 (48.9%) | 0.21 |
| Type 2 Diabetes | 592 (21.8%) | 3,320 (23.3%) | 0.12 |
| Heart Failure | 559 (20.6%) | 3,420 (24.0%) | <0.001 |
| Chronic Liver Disease | 366 (13.5%) | 2,280 (16.0%) | 0.002 |
| ***Laboratory Values*** |  |  |  |
| Hemoglobin (g/dL), Mean ± SD | 10.8 ± 2.0 | 9.9 ± 2.3 | <0.001 |
| Platelet Count (×10⁹/L), Mean ± SD | 204.5 ± 114.7 | 178.2 ± 126.4 | <0.001 |
| Creatinine (mg/dL), Mean ± SD | 2.1 ± 1.5 | 2.8 ± 2.0 | <0.001 |
| ***Interventions, n (%)*** |  |  |  |
| Mechanical Ventilation | 2,410 (88.9%) | 13,100 (92.1%) | <0.001 |
| Vasopressor Use | 1,980 (73.0%) | 11,820 (83.1%) | <0.001 |
| CRRT | 57 (2.1%) | 850 (6.0%) | <0.001 |
| ***AKI Stage, n (%)*** |  |  |  |
| Stage 1 | 715 (26.4%) | 3,200 (22.5%) | <0.001 |
| Stage 2 | 1,490 (54.9%) | 7,300 (51.3%) | 0.002 |
| Stage 3 | 507 (18.7%) | 3,726 (26.2%) | <0.001 |
| ***Outcomes*** |  |  |  |
| ICU LOS (days), Median [IQR] | 4.2 [2.8-7.1] | 2.1 [1.0-4.3] | <0.001 |
| 30-Day Mortality, n (%) | 840 (31.0%) | 5,980 (42.1%) | <0.001 |
| 90-Day Mortality, n (%) | 1,120 (41.3%) | 7,850 (55.2%) | <0.001 |
