# Supplementary material for: The non-linear association between creatinine-to-albumin ratio and medium-term mortality in patients with sepsis accompanied by acute kidney injury in the intensive care unit: a retrospective study based on the MIMIC database and external validation
Source: Front Cell Infect Microbiol. 2025 Dec 5;15:1602921. doi: 10.3389/fcimb.2025.1602921 (PMC12715007; doi:10.3389/fcimb.2025.1602921)
Supplement: Supplementary file 7 [file Table2.docx]

| **Supplementary Table S2. Other characteristics and outcomes of participants categorized by CAR** | | | | | | | | | | | | | | |
| --- | --- | --- | --- | --- | --- | --- | --- | --- | --- | --- | --- | --- | --- | --- |
| Variables | Total (n=2,712) | Q1 (n=673) | | | | Q2 (n=677) | | | | Q3 (n=681) | Q4 (n=681) | Statistic | *P* | |
|  |  |  |  |  |  |  |  |  |  |  |  |  |  |  |
| ***Characteristics*** |  |  |  | |  | | |  | |  |  | | | |
| Language, n(%) |  |  | | | |  | | | |  |  | χ²=0.72 | 0.868 | |
| Other | 276 (10.18) | 70 (10.40) | | | | 65 (9.60) | | | | 74 (10.87) | 67 (9.84) |  |  | |
| English | 2436 (89.82) | 603 (89.60) | | | | 612 (90.40) | | | | 607 (89.13) | 614 (90.16) |  |  | |
| Marital Status, n(%) |  |  | | | |  | | | |  |  | χ²=14.36 | 0.278 | |
| Divorced | 210 (7.74) | 52 (7.73) | | | | 57 (8.42) | | | | 56 (8.22) | 45 (6.61) |  |  | |
| Married | 1090 (40.19) | 243 (36.11) | | | | 293 (43.28) | | | | 274 (40.23) | 280 (41.12) |  |  | |
| Unknown | 391 (14.42) | 114 (16.94) | | | | 91 (13.44) | | | | 96 (14.10) | 90 (13.22) |  |  | |
| Single | 741 (27.32) | 199 (29.57) | | | | 166 (24.52) | | | | 183 (26.87) | 193 (28.34) |  |  | |
| Widowed | 280 (10.32) | 65 (9.66) | | | | 70 (10.34) | | | | 72 (10.57) | 73 (10.72) |  |  | |
| Laboratory parameters |  |  | |  | | |  | |  | |  | |  | |
| Lymphocytes (×109/L) | 1.37 ± 1.74 | 1.26 ± 0.81 | | | | 1.41 ± 1.37 | | | | 1.65 ± 3.00 | 1.19 ± 1.15 | | F=1.67 | 0.172 |
| Globulin (mg/dL) | 2.54 ± 0.86 | 2.61 ± 0.35 | | | | 2.57 ± 0.62 | | | | 2.64 ± 0.88 | 2.29 ± 1.39 | | F=0.61 | 0.612 |
| Triglyceride (mg/dL) | 152.09 ± 157.59 | 126.85 ± 84.08 | | | | 152.98 ± 132.67 | | | | 179.52 ± 236.54 | 158.27 ± 157.96 | | F=2.06 | 0.104 |
| ***Treatment*** |  |  | | | |  | | | |  |  |  |  | |
| Ventilation (n(%)) |  |  | | | |  | | | |  |  | χ²=1.83 | 0.608 | |
| Yes | 2410 (88.86) | 598 (88.86) | | | | 610 (90.10) | | | | 604 (88.69) | 598 (87.81) |  |  | |
| No | 302 (11.14) | 75 (11.14) | | | | 67 (9.90) | | | | 77 (11.31) | 83 (12.19) |  |  | |
| CRRT (days) | 4.28 ± 5.36 | 5.00 ± 5.66 | | | | NaN ± NA | | | | 10.00 ± 2.83 | 4.04 ± 5.37 | F=1.22 | 0.304 | |
| ***Comorbidity*** |  |  | | | |  | | | |  |  |  |  | |
| Myocardial Infarction (n(%)) |  |  | | | |  | | | |  |  | χ²=4.84 | 0.184 | |
| No | 2555 (94.21) | 638 (94.80) | | | | 641 (94.68) | | | | 630 (92.51) | 646 (94.86) |  |  | |
| Yes | 157 (5.79) | 35 (5.20) | | | | 36 (5.32) | | | | 51 (7.49) | 35 (5.14) |  |  | |
| Malignant Tumor (n(%)) |  |  | | | |  | | | |  |  | χ²=6.76 | 0.080 | |
| No | 2336 (86.14) | 595 (88.41) | | | | 585 (86.41) | | | | 587 (86.20) | 569 (83.55) |  |  | |
| Yes | 376 (13.86) | 78 (11.59) | | | | 92 (13.59) | | | | 94 (13.80) | 112 (16.45) |  |  | |
| Hepatitis (n(%)) |  |  | | | |  | | | |  |  | χ²=3.65 | 0.301 | |
| No | 2561 (94.43) | 636 (94.50) | | | | 630 (93.06) | | | | 646 (94.86) | 649 (95.30) |  |  | |
| Yes | 151 (5.57) | 37 (5.50) | | | | 47 (6.94) | | | | 35 (5.14) | 32 (4.70) |  |  | |
| Stroke (n(%)) |  |  | | | |  | | | |  |  | χ²=0.40 | 0.941 | |
| No | 2487 (91.70) | 615 (91.38) | | | | 619 (91.43) | | | | 628 (92.22) | 625 (91.78) |  |  | |
| Yes | 225 (8.30) | 58 (8.62) | | | | 58 (8.57) | | | | 53 (7.78) | 56 (8.22) |  |  | |
| GCS score (score) | 13.47 ± 2.85 | 13.53 ± 2.67 | | | | 13.41 ± 2.93 | | | | 13.33 ± 3.05 | 13.60 ± 2.72 | F=0.95 | 0.415 | |
| CAR: Q1 (Quartile 1), Q2 (Quartile 2), Q3 (Quartile 3) and Q4 (Quartile 4). Continuous variables are expressed as the median and interquartile range. Counting data are presented as numbers and percentages. The medical condition was defined based on the ICD-9 code. F: ANOVA, χ²: Chi-square test; SD: standard deviation; CRRT, continuous renal replacement therapy; GCS, Glasgow Coma Scale. | | | | | | | | | | | | | | |
