# Supplementary material for: The non-linear association between creatinine-to-albumin ratio and medium-term mortality in patients with sepsis accompanied by acute kidney injury in the intensive care unit: a retrospective study based on the MIMIC database and external validation
Source: Front Cell Infect Microbiol. 2025 Dec 5;15:1602921. doi: 10.3389/fcimb.2025.1602921 (PMC12715007; doi:10.3389/fcimb.2025.1602921)
Supplement: Supplementary file 8 [file Table3.docx]

| **Supplementary Table S3. Subgroup analysis of CAR and mortality by infection type** | | | | | | |
| --- | --- | --- | --- | --- | --- | --- |
| **Infection Type** | **Hospital Mortality** |  |  | **ICU Mortality** |  |  |
|  | **OR (95% CI)** | **P-value** | **P for interaction** | **OR (95% CI)** | **P-value** | **P for interaction** |
| **Pulmonary** | 1.42 (1.18-1.71) | <0.001 | 0.324 | 1.38 (1.12-1.70) | 0.002 | 0.287 |
| **Abdominal** | 1.38 (1.12-1.70) | 0.002 |  | 1.35 (1.08-1.69) | 0.008 |  |
| **Urinary tract** | 1.51 (1.22-1.87) | <0.001 |  | 1.47 (1.17-1.85) | 0.001 |  |
| **Bloodstream** | 1.45 (1.16-1.81) | 0.001 |  | 1.42 (1.12-1.80) | 0.004 |  |
| **Other infections** | 1.39 (1.14-1.69) | 0.001 |  | 1.36 (1.10-1.68) | 0.005 |  |

Note: CAR, creatinine-to-albumin ratio; OR, odds ratio; CI, confidence interval
