# Supplementary material for: The non-linear association between creatinine-to-albumin ratio and medium-term mortality in patients with sepsis accompanied by acute kidney injury in the intensive care unit: a retrospective study based on the MIMIC database and external validation
Source: Front Cell Infect Microbiol. 2025 Dec 5;15:1602921. doi: 10.3389/fcimb.2025.1602921 (PMC12715007; doi:10.3389/fcimb.2025.1602921)
Supplement: Supplementary file 9 [file Table4.docx]

| **Supplementary Table S4. External Validation of CAR Prognostic Performance** | | | |
| --- | --- | --- | --- |
| **Characteristic** | **Derivation Cohort (n=2,712)** | **Validation Cohort (n=412)** | **P-value** |
| **Demographics** |  |  |  |
| Age (years), Mean ± SD | 63.3 ± 16.6 | 64.1 ± 15.8 | 0.32 |
| Male Sex, n (%) | 1,456 (53.7%) | 225 (54.6%) | 0.75 |
| **Disease Severity** |  |  |  |
| SOFA Score, Mean ± SD | 7.6 ± 4.1 | 7.9 ± 4.2 | 0.18 |
| APSIII Score, Mean ± SD | 57.1 ± 23.3 | 58.3 ± 22.7 | 0.41 |
| **Laboratory Parameters** |  |  |  |
| CAR, Mean ± SD (mg/dL) | 1.2 ± 0.8 | 1.3 ± 0.9 | 0.09 |
| Lactate, Mean ± SD (mmol/L) | 2.9 ± 1.9 | 3.1 ± 2.0 | 0.07 |
| **Outcomes** |  |  |  |
| 30-Day Hospital Mortality, n (%) | 840 (31.0%) | 135 (32.8%) | 0.48 |
| 30-Day ICU Mortality, n (%) | 745 (27.5%) | 118 (28.6%) | 0.65 |
| **CAR Prognostic Performance** | HR (95% CI) | HR (95% CI) |  |
| Hospital Mortality (adjusted) | 1.16 (1.00-1.35) | 1.21 (1.02-1.43) | - |
| ICU Mortality (adjusted) | 1.18 (1.00-1.39) | 1.19 (1.01-1.41) | - |
| **Discriminatory Power (AUC)** | AUC (95% CI) | AUC (95% CI) |  |
| Hospital Mortality | 0.69 (0.66-0.72) | 0.68 (0.62-0.74) | - |
| ICU Mortality | 0.67 (0.64-0.70) | 0.66 (0.60-0.72) | - |
| **CAR ≥1.2 mg/dL Performance** | Sensitivity/Specificity | Sensitivity/Specificity |  |
| Hospital Mortality | 72%/64% | 70%/62% | - |
| ICU Mortality | 69%/63% | 67%/61% | - |

Note: CAR, creatinine-to-albumin ratio; HR, hazard ratio; CI, confidence interval; AUC, area under the receiver operating characteristic curve; SOFA, Sequential Organ Failure Assessment; APSIII, Acute Physiology Score III. Adjusted models included age, gender, SOFA score, and lactate levels.
