# Supplementary material for: The non-linear association between creatinine-to-albumin ratio and medium-term mortality in patients with sepsis accompanied by acute kidney injury in the intensive care unit: a retrospective study based on the MIMIC database and external validation
Source: Front Cell Infect Microbiol. 2025 Dec 5;15:1602921. doi: 10.3389/fcimb.2025.1602921 (PMC12715007; doi:10.3389/fcimb.2025.1602921)
Supplement: Supplementary file 10 [file Table5.docx]

| **Supplementary Table S5. Detailed Baseline Characteristics of External Validation Cohort by CAR Threshold** | | | | |
| --- | --- | --- | --- | --- |
| Characteristic | CAR <1.2 mg/dL (n=248) | CAR ≥1.2 mg/dL (n=164) | P-value | Standardized Difference |
| **Demographics** |  |  |  |  |
| Age (years), Mean ± SD | 62.3 ± 14.7 | 66.8 ± 17.1 | 0.004 | 0.29 |
| Male Sex, n (%) | 132 (53.2%) | 93 (56.7%) | 0.48 | 0.07 |
| BMI (kg/m²), Mean ± SD | 26.8 ± 5.2 | 25.3 ± 6.1 | 0.008 | 0.26 |
| **Clinical Severity** |  |  |  |  |
| SOFA Score, Mean ± SD | 6.8 ± 3.5 | 9.5 ± 4.6 | <0.001 | 0.67 |
| APACHE II, Mean ± SD | 18.3 ± 6.2 | 24.1 ± 7.8 | <0.001 | 0.82 |
| qSOFA ≥2, n (%) | 89 (35.9%) | 98 (59.8%) | <0.001 | 0.49 |
| **Laboratory Values** |  |  |  |  |
| Creatinine (mg/dL), Mean ± SD | 1.4 ± 0.5 | 3.2 ± 1.8 | <0.001 | 1.28 |
| Albumin (g/dL), Mean ± SD | 3.5 ± 0.6 | 2.4 ± 0.5 | <0.001 | 2.01 |
| Lactate (mmol/L), Mean ± SD | 2.3 ± 1.2 | 4.2 ± 2.3 | <0.001 | 1.02 |
| CRP (mg/L), Mean ± SD | 85.3 ± 42.1 | 142.6 ± 67.8 | <0.001 | 1.00 |
| Procalcitonin (ng/mL), Median [IQR] | 2.1 [0.8-5.3] | 8.7 [3.2-18.4] | <0.001 | 0.89 |
| **Interventions** |  |  |  |  |
| Mechanical Ventilation, n (%) | 198 (79.8%) | 152 (92.7%) | <0.001 | 0.37 |
| Vasopressor Use, n (%) | 156 (62.9%) | 142 (86.6%) | <0.001 | 0.56 |
| CRRT, n (%) | 8 (3.2%) | 27 (16.5%) | <0.001 | 0.46 |
| **Outcomes** |  |  |  |  |
| 28-Day Hospital Mortality, n (%) | 52 (21.0%) | 83 (50.6%) | <0.001 | 0.65 |
| ICU LOS (days), Median [IQR] | 6 [4-10] | 11 [7-18] | <0.001 | 0.71 |
| Hospital LOS (days), Median [IQR] | 14 [9-21] | 9 [5-15] | <0.001 | 0.63 |
