# Supplementary material for: The non-linear association between creatinine-to-albumin ratio and medium-term mortality in patients with sepsis accompanied by acute kidney injury in the intensive care unit: a retrospective study based on the MIMIC database and external validation
Source: Front Cell Infect Microbiol. 2025 Dec 5;15:1602921. doi: 10.3389/fcimb.2025.1602921 (PMC12715007; doi:10.3389/fcimb.2025.1602921)
Supplement: Supplementary file 11 [file Table6.docx]

| **Supplementary Table S6. Time-Dependent ROC Analysis of CAR for Mortality Prediction** | | | | |
| --- | --- | --- | --- | --- |
| **Time Point** | **CAR AUC (95% CI)** | **Lactate AUC (95% CI)** | **SOFA AUC (95% CI)** | **Combined Model AUC (95% CI)** |
| 7-Day Mortality | 0.75 (0.69-0.81) | 0.73 (0.67-0.79) | 0.76 (0.70-0.82) | 0.82 (0.77-0.87) |
| 14-Day Mortality | 0.72 (0.67-0.77) | 0.70 (0.65-0.75) | 0.74 (0.69-0.79) | 0.79 (0.75-0.83) |
| 21-Day Mortality | 0.70 (0.65-0.75) | 0.68 (0.63-0.73) | 0.72 (0.67-0.77) | 0.77 (0.73-0.81) |
| 28-Day Mortality | 0.68 (0.63-0.73) | 0.66 (0.61-0.71) | 0.70 (0.65-0.75) | 0.75 (0.71-0.79) |
