# Supplementary material for: The non-linear association between creatinine-to-albumin ratio and medium-term mortality in patients with sepsis accompanied by acute kidney injury in the intensive care unit: a retrospective study based on the MIMIC database and external validation
Source: Front Cell Infect Microbiol. 2025 Dec 5;15:1602921. doi: 10.3389/fcimb.2025.1602921 (PMC12715007; doi:10.3389/fcimb.2025.1602921)
Supplement: Supplementary file 12 [file Table7.docx]

| **Supplementary Table S7. Subgroup Analysis in External Validation Cohort** | | | | |
| --- | --- | --- | --- | --- |
| **Subgroup** | **Patients (n)** | **Hospital Mortality HR (95% CI)** | **P-value** | **P for interaction** |
| **Overall** | 412 | 1.21 (1.02-1.43) | 0.032 | - |
| **Age** |  |  |  | 0.045 |
| <65 years | 218 | 1.32 (1.08-1.61) | 0.007 |  |
| ≥65 years | 194 | 1.09 (0.86-1.38) | 0.48 |  |
| **Septic Shock** |  |  |  | 0.021 |
| No | 158 | 1.42 (1.12-1.80) | 0.004 |  |
| Yes | 254 | 1.08 (0.87-1.34) | 0.49 |  |
| **Infection Source** |  |  |  | 0.128 |
| Pulmonary | 186 | 1.25 (1.01-1.55) | 0.043 |  |
| Abdominal | 98 | 1.18 (0.87-1.60) | 0.29 |  |
| Urinary Tract | 78 | 1.31 (0.95-1.81) | 0.10 |  |
| Bloodstream | 50 | 1.45 (0.98-2.15) | 0.064 |  |
| **AKI Stage** |  |  |  | 0.038 |
| Stage 1 | 128 | 1.52 (1.15-2.01) | 0.003 |  |
| Stage 2 | 186 | 1.18 (0.93-1.50) | 0.18 |  |
| Stage 3 | 98 | 1.03 (0.75-1.41) | 0.87 |  |
