# Supplementary material for: The non-linear association between creatinine-to-albumin ratio and medium-term mortality in patients with sepsis accompanied by acute kidney injury in the intensive care unit: a retrospective study based on the MIMIC database and external validation
Source: Front Cell Infect Microbiol. 2025 Dec 5;15:1602921. doi: 10.3389/fcimb.2025.1602921 (PMC12715007; doi:10.3389/fcimb.2025.1602921)
Supplement: Supplementary file 13 [file Table8.docx]

| **Supplementary Table S8. Comparison of CAR with Established Biomarkers** | | | | | | |
| --- | --- | --- | --- | --- | --- | --- |
| **Biomarker/Score** | **Hospital Mortality AUC (95% CI)** | **P-value** | **ICU Mortality AUC (95% CI)** | **P-value** | **IDI (P-value)** | **NRI (P-value)** |
| **CAR** | 0.69 (0.66-0.72) | Reference | 0.67 (0.64-0.70) | Reference | - | - |
| **Lactate** | 0.71 (0.68-0.74) | 0.42 | 0.70 (0.67-0.73) | 0.38 | 0.008 (0.35) | 0.08 (0.28) |
| **SOFA Score** | 0.73 (0.70-0.76) | 0.28 | 0.72 (0.69-0.75) | 0.24 | 0.015 (0.18) | 0.12 (0.15) |
| **AKI Stage** | 0.65 (0.62-0.68) | 0.04 | 0.63 (0.60-0.66) | 0.03 | 0.025 (0.02) | 0.18 (0.01) |
| **Combined Models** |  |  |  |  |  |  |
| CAR + SOFA | 0.75 (0.72-0.78) | <0.001* | 0.74 (0.71-0.77) | <0.001* | 0.021 (0.038) | 0.15 (0.026) |
| CAR + Lactate | 0.74 (0.71-0.77) | 0.002* | 0.73 (0.70-0.76) | 0.003* | 0.018 (0.045) | 0.13 (0.039) |
| CAR + SOFA + Lactate | 0.77 (0.74-0.80) | <0.001* | 0.76 (0.73-0.79) | <0.001* | 0.035 (0.008) | 0.22 (0.012) |

*Note: CAR, creatinine-to-albumin ratio; AUC, area under the receiver operating characteristic curve; IDI, integrated discrimination improvement; NRI, net reclassification improvement; SOFA, Sequential Organ Failure Assessment; AKI, acute kidney injury. *P-value for comparison with CAR alone.*
